# Methylation of KSHV vCyclin by PRMT5 contributes to cell cycle progression and cell proliferation

**DOI:** 10.1371/journal.ppat.1012535

**Published:** 2024-09-10

**Authors:** Danping Niu, Yuanming Ma, Pengyu Ren, Sijia Chang, Chenhui Li, Yong Jiang, Chunyan Han, Ke Lan

**Affiliations:** 1 State Key Laboratory of Virology, College of Life Sciences, Wuhan University, Wuhan, China; 2 Department of Infectious Diseases, Frontier Science Center for Immunology and Metabolism, Medical Research Institute, Zhongnan Hospital of Wuhan University, Wuhan University, Wuhan, China; 3 Taikang Center for Life and Medical Sciences, Wuhan University, Wuhan, China; Florida State University, UNITED STATES OF AMERICA

## Abstract

Kaposi’s sarcoma-associated herpesvirus (KSHV) is a double-stranded DNA virus that encodes numerous cellular homologs, including cyclin D, G protein-coupled protein, interleukin-6, and macrophage inflammatory proteins 1 and 2. KSHV vCyclin encoded by ORF72, is the homolog of cellular cyclinD2. KSHV vCyclin can regulate virus replication and cell proliferation by constitutively activating cellular cyclin-dependent kinase 6 (CDK6). However, the regulatory mechanism of KSHV vCyclin has not been fully elucidated. In the present study, we identified a host protein named protein arginine methyltransferase 5 (PRMT5) that interacts with KSHV vCyclin. We further demonstrated that PRMT5 is upregulated by latency-associated nuclear antigen (LANA) through transcriptional activation. Remarkably, knockdown or pharmaceutical inhibition (using EPZ015666) of PRMT5 inhibited the cell cycle progression and cell proliferation of KSHV latently infected tumor cells. Mechanistically, PRMT5 methylates vCyclin symmetrically at arginine 128 and stabilizes vCyclin in a methyltransferase activity-dependent manner. We also show that the methylation of vCyclin by PRMT5 positively regulates the phosphorylate retinoblastoma protein (pRB) pathway. Taken together, our findings reveal an important regulatory effect of PRMT5 on vCyclin that facilitates cell cycle progression and proliferation, which provides a potential therapeutic target for KSHV-associated malignancies.

## Author summary

KSHV vCyclin contributes to KSHV latency by promoting cell cycle progression and proliferation, but the regulation and posttranslational modifications (PTMs) of vCyclin have not been fully elucidated. Here, we report that PRMT5, a novel vCyclin binding partner, is significantly upregulated by LANA. Importantly, PRMT5 methylates vCyclin symmetrically and stabilizes vCyclin in a methyltransferase activity-dependent manner, thereby promoting cell cycle progression and proliferation of KSHV latently infected tumor cells. We also report that the methylation of vCyclin positively regulates the pRB pathway. Taken together, the findings indicate that PRMT5 plays an essential role in regulating vCyclin to facilitate cell cycle progression and proliferation and may serve as a therapeutic target for KSHV-associated diseases.

## Introduction

Kaposi’s sarcoma-associated herpesvirus (KSHV), also referred to as human herpesvirus 8, is a tumorigenic human DNA γ herpesvirus. KSHV is etiologically linked to three human malignancies, including Kaposi’s sarcoma (KS), primary effusion lymphoma (PEL), and multicentric Castleman’s disease (MCDs) [1–3]. Like other herpesviruses, KSHV has a two-phase life cycle consisting of a latent infection phase and a lytic replication phase [4]. In most KSHV-infected pathologic tissues and cell lines, the virus is in a state of latent infection, and only a small number of cells exhibit spontaneous lytic reactivation [5, 6]. Furthermore, latent infection is defined by the absence of observable viral particle production and expression of only a small number of viral genes, such as ORF71 (viral Fas-associated death domain-like IL-1-converting enzyme inhibitory protein, vFLIP), ORF72 (viral Cyclin, vCyclin), ORF73 (latency-associated nuclear antigen, LANA), K12 (kaposins), and microRNAs. These genes function in growth, proliferation, resistance to apoptosis, angiogenesis and inflammation, and contribute to limitless replicative potential [5, 7]. It is imperative for the success of a KSHV latency program that the infected cells survive and continue to multiply [4]. Thus, determining the molecular mechanisms by which KSHV latently infected cells survive and proliferate is vital to understanding the pathogenesis of KSHV.

KSHV-encoded vCyclin has been reported to activate cellular CDK6, and accelerate the G1-S transition of the cell cycle, causing DNA damage, apoptosis, and autophagy. It is also thought to have carcinogenic potential [8]. vCyclin forms an active kinase complex with CDK6 and shares structural similarities with cellular D-type cyclin [9,10]. The binding of vCyclin activates CDK6 to phosphorylate retinoblastoma protein (pRB), histone H1 [11], p27KIP1 [12], p21CIP1, ORC-1, CDC6, caldesmon, Bcl-2 [13], CDC25a, and Nucleophosmin [14,15]. Currently, it is known that the genomes of eight distinct viruses encode ORFs with cyclin homologs. All of these viral cyclins have a conserved region known as the "cyclin box" [16]. Many viral cyclins are homologous to cellular cyclin D, which can functionally substitute for them in their absence, thus ensuring a favorable environment for the survival of viruses and the development of tumors [17]. Therefore, viral cyclins may cause tissue hyperproliferation in infected hosts, which may occasionally progress to clonal malignancies depending on the circumstances [8,18–21].

Protein arginine methyltransferases (PRMTs) are a family of nine enzymes that add a methyl group to arginine residues on their target proteins from S-adenosyl methionine. PRMTs are divided into three groups according to the following methylation patterns: monomethylation (MMA), symmetric demethylation (SDMA), and asymmetric demethylation (ADMA) [22]. PRMT5 is a major disymmetric arginine methyltransferase in mammalian cells and might be a vital therapeutic target molecule for cancer treatment [23]. It performs symmetric dimethylation on histones (i.e., H3 and H4) as well as nonhistone proteins (e.g., retinoblastoma [Rb], p53, and Sm proteins of the spliceosome), thereby participating in multiple oncogenic processes via transcription, splicing, translation, signal transduction, DNA damage and repair, and cell cycle regulation [24–26]. In addition to playing a crucial role in providing support for cell proliferation and cell cycle progression, PRMT5 promotes anchorage-independent growth and mediates the development of neoplastic cells induced by cyclin D1/CDK4 [27,28]. In addition, PRMT5 promotes the Cyclin E1/Cdk2 pair and G1/S progression, correlating with experimental autoimmune encephalomyelitis (EAE) severity [29]. Interestingly, an effective and well-tolerated treatment approach involves combining CDK4/6 inhibition with PRMT5 inhibition [30].

While substantial research has been conducted on how vCyclin activates CDK and phosphorylates RB [31], the regulatory mechanisms and the PTMs of vCyclin itself remain unclear. Here, we identified PRMT5 as a novel vCyclin-binding host protein. We further demonstrated that LANA can upregulate PRMT5 through transcriptional activation in cells latently infected with KSHV. Mechanistically, PRMT5 symmetrically methylates and stabilizes vCyclin, which promotes cell cycle progression and cell proliferation. In addition, the methylation of vCyclin by PRMT5 positively regulates the pRB pathway.

## Results

### PRMT5 interacts with KSHV vCyclin

**Fig 1:**
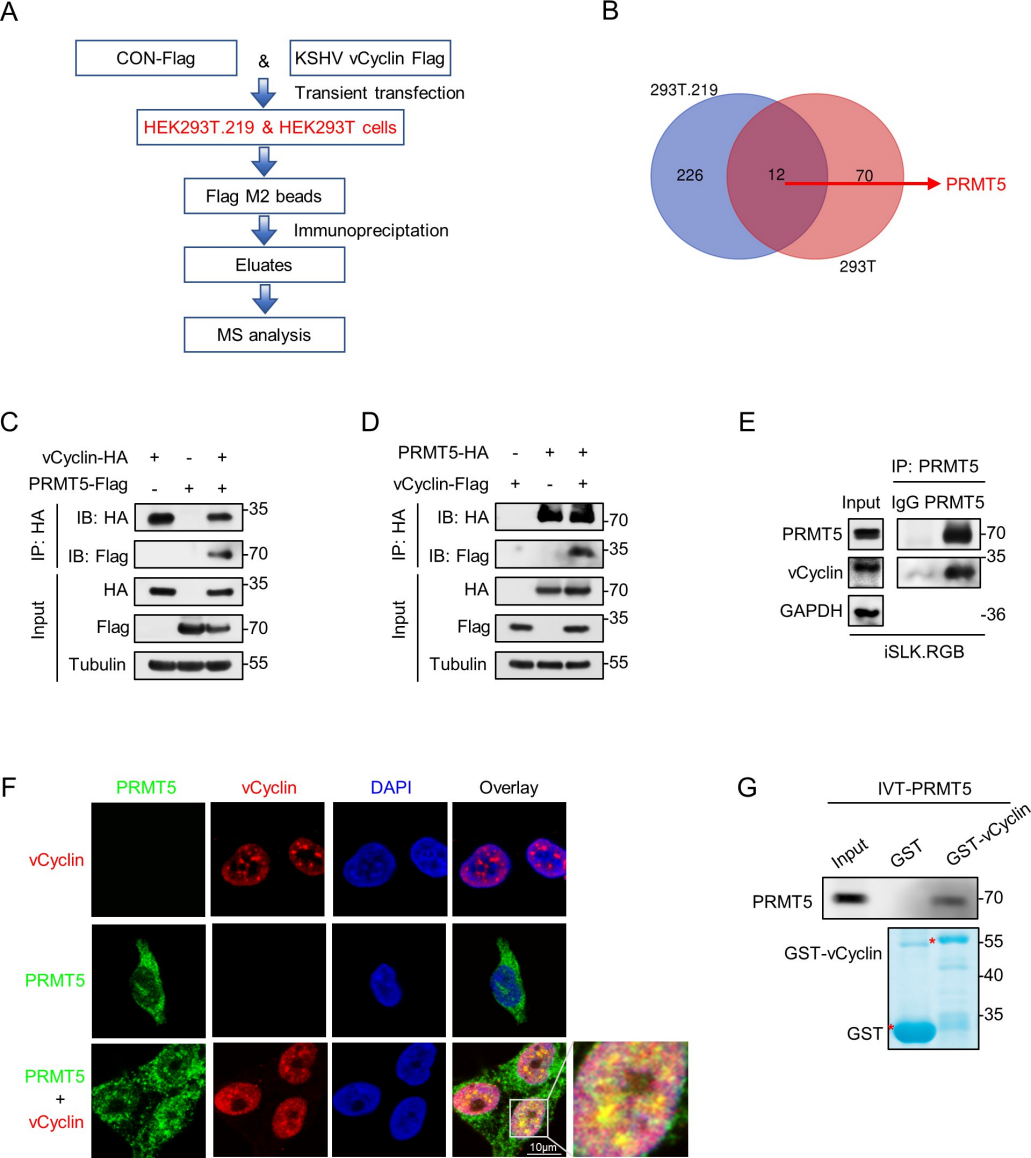
PRMT interacts with KSHV vCyclin. (A) Schematic strategy showing purification and identification of vCyclin binding proteins via immunoprecipitation (IP) assay. Flag-tagged vCyclin plasmid was transfected into HEK293T and HEK293T.219 cells. The equivalent empty vector was transfected as a control. Cell lysates were performed for affinity purification by immunoprecipitation with FLAG M2 beads. The purified elutes were analyzed by MS. (B) Venn diagram describing the overlap of all proteins identified via mass spectrometry from (A). (C-D) Co-IP analysis of the interaction between PRMT5 and vCyclin in HEK293T cells. HEK293T cells were first cotransfected with Flag-PRMT5 and HA-vCyclin plasmids (C) and then cotransfected with HA-PRMT5 and Flag-vCyclin plasmids (D). IP assays were done by anti-HA antibodies to show the interaction between PRMT5 and vCyclin. (E) Physical interaction between endogenous PRMT5 and vCyclin in iSLK.RGB cells. Cell lysates from iSLK.RGB were subjected to immunoprecipitation with anti-PRMT5 antibody or rabbit IgG controls. Then, IB analysis of WCLs and immunoprecipitation (IP) products derived from iSLK.RGB was performed. (F) Colocalization of endogenous vCyclin and PRMT5 in iSLK.BAC16 cells. The cells were fixed and probed with anti-PRMT5 rabbit antibody and anti-vCyclin rat antibody, followed by incubation with secondary antibodies conjugated to Alexa Fluor 568 (red) or Alexa Fluor 647 (green), DAPI (blue). Colocalization was viewed by a Leica SP8 laser confocal microscopy. Scale bars represent 10 μm. (G) In vitro interaction between PRMT5 and vCyclin via GST pull-down assay. Purified GST or GST-fused vCyclin were incubated with GST-tagged agarose beads for 4 hours. Then purified beads were incubated with equivalent in vitro translated IVT–PRMT5 and pulled-down protein complexes were subjected to western blotting detection (upper panel) and Coomassie Blue staining (lower panel). The input lane represents 1/10 of IVT-PRMT5 programmed into the GST pull-down reaction.

During the latent phase, KSHV expresses a limited number of viral genes, including ORF73, ORF72, ORF71, and ORFK12, along with 25 mature microRNAs [32]. vCyclin, encoded by ORF72, is the homolog of cellular cyclinD2. However, vCyclin exhibits markedly distinct characteristics compared to cyclinD2. For example, the half-life of vCyclin (~6 hours) was much longer than that of cellular cyclin D2 (~30 minutes) [33]. To gain insight into the molecular mechanism by which vCyclin is regulated, we screened for vCyclin binding partners via Flag-tag-based affinity purification and mass spectrometric (MS) analyses (Fig 1A). Flag-tagged vCyclin and equivalent Flag vector plasmids were transfected into human embryonic kidney (HEK)293T.219 (KSHV-positive) and HEK293T (KSHV-negative) cells. Cell lysates were performed for affinity purification by immunoprecipitation with FLAG M2 beads. A few novel proteins were identified by peptide correlation, and the results were summarized in S1 and S2 Tables. With proteins identified by mass spectrometry, we constructed a Venn diagram, which illustrates that 12 proteins are enriched in both HEK293T.219 and HEK293T cells (Fig 1B). The results were summarized in S3 Table. Among 12 candidate proteins, 5 genes related to tumorigenesis were further validated the interaction with vCyclin. Among the 5 valiated binding factors, PRMT5 (~70-kDa band) is a well-established oncogene involved in numerous malignancies and is crucial for regulating the cell cycle and cell proliferation. Consequently, we ultimately selected enzymatic PRMT5 as the focus of our investigation. The interaction between PRMT5 and vCyclin was confirmed by coimmunoprecipitation (co-IP) assays showing that PRMT5 formed complexes with vCyclin in HEK293T cells (Fig 1C and 1D). To further confirm the endogenous interaction between PRMT5 and vCyclin, we performed co-IP assays in iSLK.RGB cells (Fig 1E). co-IP assays revealed the formation of complexes between vCyclin and PRMT5 in KSHV-infected cells. Moreover, the endogenous distribution and association of vCyclin with PRMT5 were examined via confocal microscopy in iSLK.BAC16 cells. As shown in Fig 1F, the results demonstrated that endogenous vCyclin and PRMT5 were colocalized in the same nuclear compartments. Furthermore, we demonstrated that PRMT5 directly interacted with vCyclin via an in vitro glutathione S-transferase (GST) pulldown assay (Fig 1G). In vitro translated PRMT5 (IVT-PRMT5) was directly bound to purified GST-vCyclin. Taken together, these results confirm that the host PRMT5 protein is a novel vCyclin- binding partner.

### LANA upregulates PRMT5 in KSHV latently infected cells through transcriptional activation

**Fig 2:**
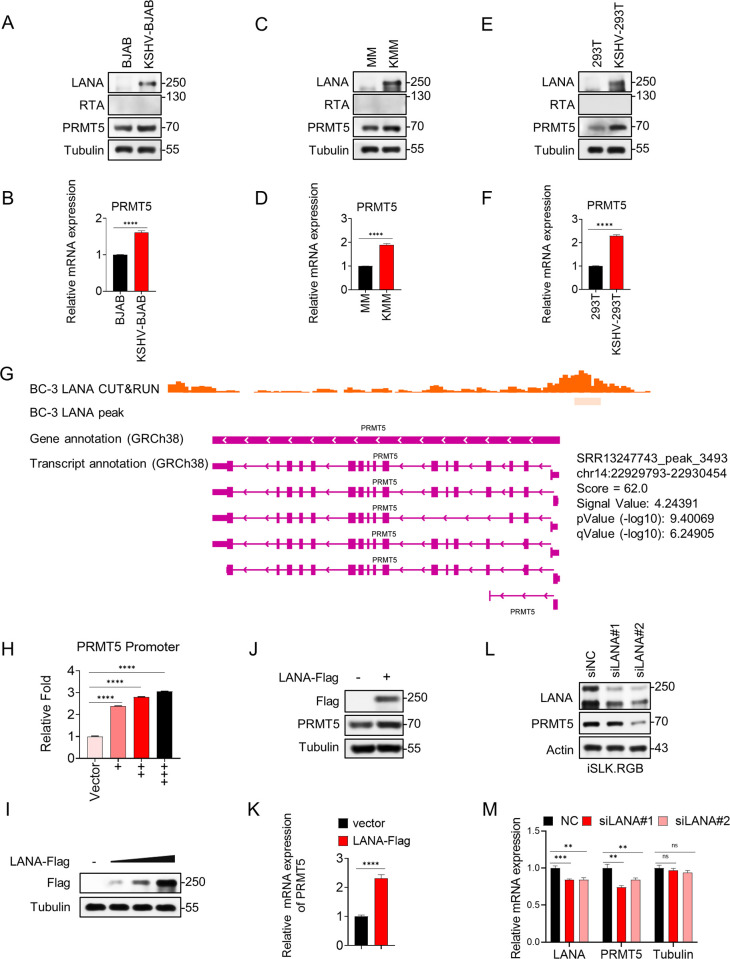
LANA upregulates PRMT5 in KSHV latently infected cells through transcriptional activation. (A-F) The expression of PRMT5 in BJAB and KSHV-BJAB cells (A-B), MM and KMM cells (C-D), HEK293T and KSHV-HEK293T cells (E-F) was measured by western blotting and quantitative real-time PCR (RT-qPCR). (G) CUT&RUN data from NCBI GEO was analyzed by IGV software. The CUT&RUN experiment was performed in BC-3 cells incubated with anti-LANA antibodies. (H-I) LANA actives PRMT5 promoter in a dose-dependent manner. (H) HEK293T cells were transfected with fixed amounts of dual-reporter plasmids and increasing amounts of LANA-expressing plasmids, as indicated. 36h post-transfection, cells were lysed and luciferase activities were analyzed. In addition, (I) lysates of cells from (H) were subjected to western blot analysis. Representative results from three biological replicates are presented. Error bars indicate SD. Data were analyzed with Student’s multiple t-tests (****p< 0.0001). (J) HEK293T cells were transfected with Flag-vector alone as a negative control or together with Flag-LANA. After 36h transfection, the cells were lysed and analyzed by western blots with indicated antibodies. (K) PRMT5 mRNA expression level in cells from (J) was analyzed by qPCR. (L-M) iSLK.RGB cells were transfected with control siRNA and two LANA-specific siRNAs. The knockdown efficiency level of LANA and the expression of PRMT5 were determined by western blotting (L) and qPCR (M). Representative results from three biological replicates are presented. Error bars indicate SD. Data were analyzed with Student’s multiple t-tests (**p < 0.01, ***p< 0.001, ****p< 0.0001).

As shown above, we identified a host protein PRMT5 that interacts directly with vCyclin. We then assessed the expression level of PRMT5 in KSHV-positive cells. Previous research strongly suggests that PRMT5 is a tumor promoter, as it is significantly overexpressed in cancers such as colon, ovarian, kidney, lung, bladder, liver, pancreatic, breast, prostate, cervical, and skin cancers [34]. In addition, KSHV is an oncogenic herpesvirus that can be involved in multiple pathways by participating in and promoting the malignant transformation of host cells [35]. Hence, we speculated that PRMT5 might be upregulated in KSHV latently infected cells. To test this possibility, we compared the protein and mRNA expression levels of PRMT5 with or without latent KSHV infection (Fig 2A–2F). The expression levels of PRMT5 were measured in KSHV-positive (KSHV-BJAB, KMM, KSHV-HEK293T) and KSHV-negative (BJAB, MM, HEK293T) cell lines. The results suggested that PRMT5 was upregulated at both the protein and mRNA levels in KSHV latently infected cells.

Previous studies have demonstrated that several cellular genes are stimulated by persistent expression of LANA, including those involved in cell proliferation and antiviral responses [36–39]. Moreover, LANA regulates the transcription of many cellular and viral genes [40]. Therefore, we speculated that LANA might be responsible for increasing PRMT5 expression by activating the PRMT5 promoter. To test this hypothesis, we first queried the NCBI GEO database and retrieved data from a cleavage under targets and release using nuclease (CUT&RUN) study conducted in BC-3 cells treated with anti-LANA antibodies[41]. Then the data was subsequently analyzed using software (IGV), revealing that LANA binds to the PRMT5 promoter (Fig 2G). Previous research has shown that PRMT5 expression is tightly controlled at the transcriptional level. In laryngeal cancer, YY1 (Yin Yang 1) binds to the promoter region of PRMT5, enhancing its transcription. This upregulation of PRMT5 promotes cancer progression and metastasis by modulating downstream targets [42]. In the context of MLL-fusion leukemia, the PAF (Polymerase Associated Factor) complex facilitates the transcriptional regulation of PRMT5, which is essential for the maintenance and progression of the leukemia [43]. On the other hand, PRMT5 can regulate its own expression indirectly through histone modification and gene activation [44]. To further explore the upregulation mechanism of PRMT5, the DNA fragment of the PRMT5 promoter (-2.0 kb to -1.0 bp) was cloned and inserted into a luciferase reporter vector and cotransfected into HEK293T cells with the LANA-expressing plasmids. A dose-dependent increase in PRMT5 promoter activity was observed with increasing amounts of LANA (Fig 2H and 2I). To further prove the hypothesis, plasmids encoding Flag-tagged LANA or empty vectors were transfected into HEK293T cells for 36 h. As expected, both the protein and mRNA expression of PRMT5 were upregulated (Fig 2J and 2K). Additionally, we knocked down endogenous LANA in iSLK.RGB cells with two specific siRNAs (siLANA#1 and siLANA#2) or transfected cells with a negative control siRNA (siNC). Consistently, the protein and mRNA expression level of PRMT5 was declined when LANA was knocked down (Fig 2L and 2M). Relative mRNA expression level of Tubulin in Fig 2M was as a negative control. Moreover, SLK cells were infected with wild-type KSHV (KSHV-WT) and LANA-depleted KSHV (KSHV-LANAstop) [45], and the expression of PRMT5 was measured. Consistent with the results in iSLK.RGB cells, the expression of PRMT5 was declined when LANA was eliminated (S1 Fig).

Based on these results, PRMT5 is upregulated in cells with latent KSHV infection, and LANA participates in this upregulation through transcriptional activation.

### Knockdown of endogenous PRMT5 inhibits cell cycle progression and cell proliferation in KSHV-infected cells

**Fig 3:**
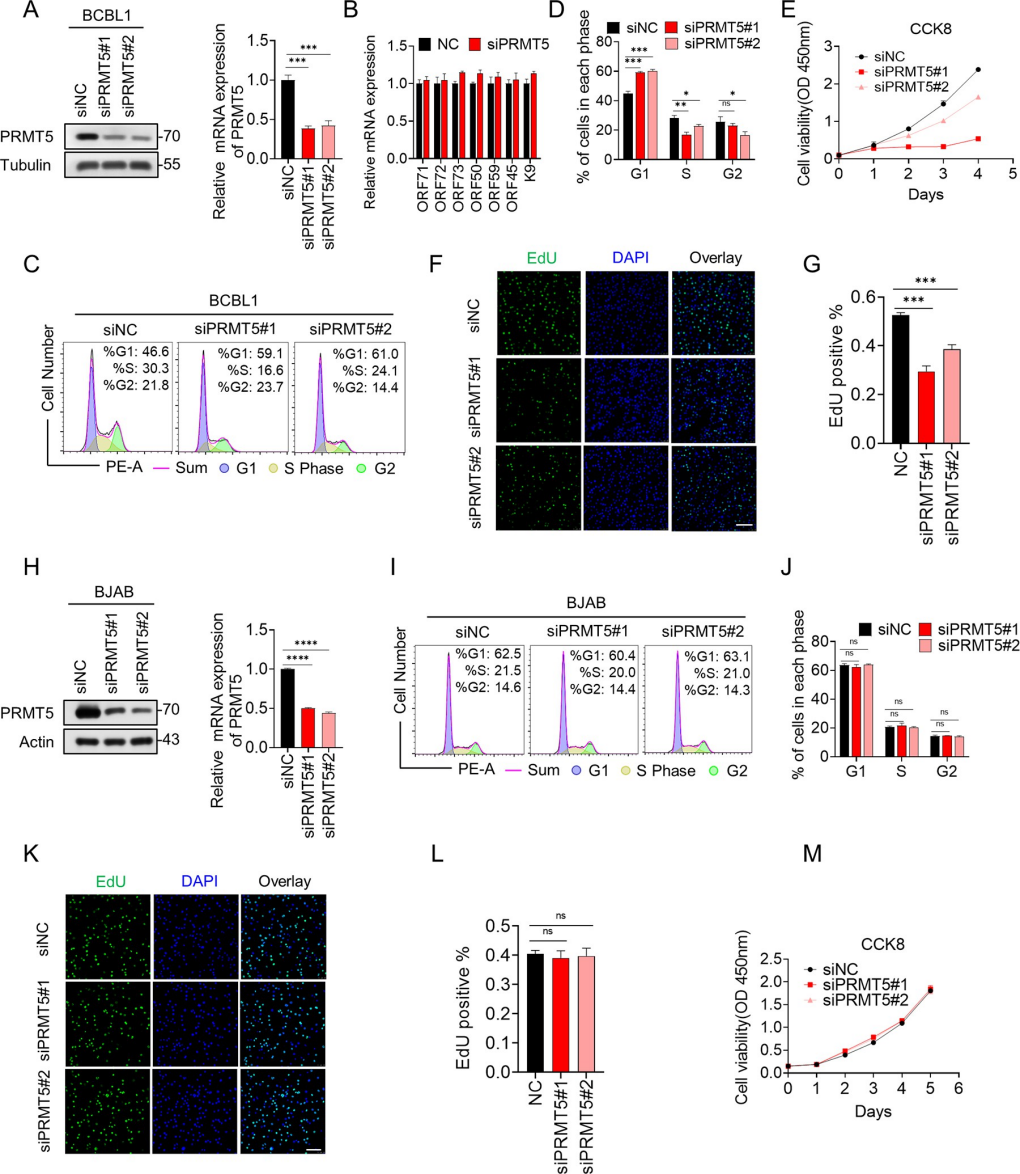
Knockdown of endogenous PRMT5 inhibits the cell cycle progression and cell proliferation in KSHV-infected cells. (A) BCBL1 cells were transfected with siRNA as indicated. 72 h post-transfection, the knockdown efficiency of PRMT5 was determined by immunoblotting and qPCR analysis. (B) The transcription level of several KSHV genes from BCBL1 with knockdown of endogenous PRMT5 was determined by qPCR analysis. (C-D) Flow cytometry was performed to analyze the cell cycle distribution. Endogenous PRMT5 in BCBL1 cells were knocked down with two specific siRNAs (siPRMT5#1 and siPRMT5#2) or transfected cells with a negative control siRNA (siNC). After 72 hours posttransfection, cells were fixed and stained for flow cytometry analysis. (E-G) The cell proliferation of BCBL1 cells was measured by CCK8 assays (E) and EdU assays (F-G) at the indicated time points. Representative immunofluorescence images showthe expression of EdU. Scale bars represent 100μm. (H) BJAB cells were transfected with siRNA as indicated. The knockdown efficiency of PRMT5 was determined by immunoblotting and qPCR analysis. (I-J) Flow cytometry was performed to analyze the cell cycle distribution in BJAB cells with transfected siNC or siPRMT5#1 or siPRMT5#2 for 72 hours. (K-M) The cell proliferation of BJAB cells was measured by CCK8 assays (M) and EdU assays (K-L) at the indicated time points. Representative immunofluorescence images show the expression of EdU. Scale bars represent 100μm. Representative results from three biological replicates are presented. Error bars indicate SD. Data were analyzed with Student’s multiple t-tests (*p < 0.05, **p< 0.01 ***p< 0.001, ****p< 0.0001).

To investigate the functional role of PRMT5 in KSHV infection, we knocked down endogenous PRMT5 in BCBL1 (KSHV positive) cells and BJAB (KSHV negative) cells with two specific siRNAs (siPRMT5#1 and siPRMT5#2) and a negative control siRNA (siNC). As shown in Fig 3A and Fig 3H, the expression of PRMT5 was significantly reduced at both the protein and mRNA levels. However, the knockdown of PRMT5 had minimal impact on the transcript levels of viral genes (ORF71, ORF72, ORF73, ORF50, ORF59, ORF45) in BCBL1 cells (Fig 3B). Importantly, flow cytometry showed that the cell cycle was arrested at the G1 phase when PRMT5 was knocked down in BCBL1 cells (Fig 3C and 3D) but not in BJAB cells (Fig 3I and 3J). Since CCK8 and EdU assays were commonly employed for detecting cell proliferation [46,47], we knocked down PRMT5 by transfecting control siRNA and two siRNAs targeting human PRMT5 in BCBL1 cells. 48h post-transfection, cells were counted and plated for CCK8 and EDU assays (Fig 3E–3G). The results showed that reducing PRMT5 inhibited the cell proliferation of BCBL1, as shown by CCK-8 (Fig 3E) and EdU assays (Fig 3F–3G). However, Knockdown of PRMT5 hardly affected cell proliferation of BJAB cells (Fig 3K–3M). To further verify the above results, we also knocked down endogenous PRMT5 in iSLK.RGB (KSHV positive) cells and SLK (KSHV negative) cells (S2 Fig). Consistent with the findings in Fig 3, PRMT5 knockdown decreased the cell cycle and proliferation of iSLK.RGB cells but had no effect on SLK cells. Furthermore, iSLK.RGB cells were treated with a pharmaceutical inhibitor (EPZ015666) of PRMT5 (S3 Fig). The data showed that the cell proliferation of iSLK.RGB cells was significantly suppressed when PRMT5 was substantially inhibited by EPZ015666. Taken together, these results convincingly suggest that knockdown of PRMT5 effectively inhibits cell cycle progression and cell proliferation in KSHV-infected cells.

### PRMT5 stabilizes vCyclin protein expression in a manner dependent on its methyltransferase activity

**Fig 4:**
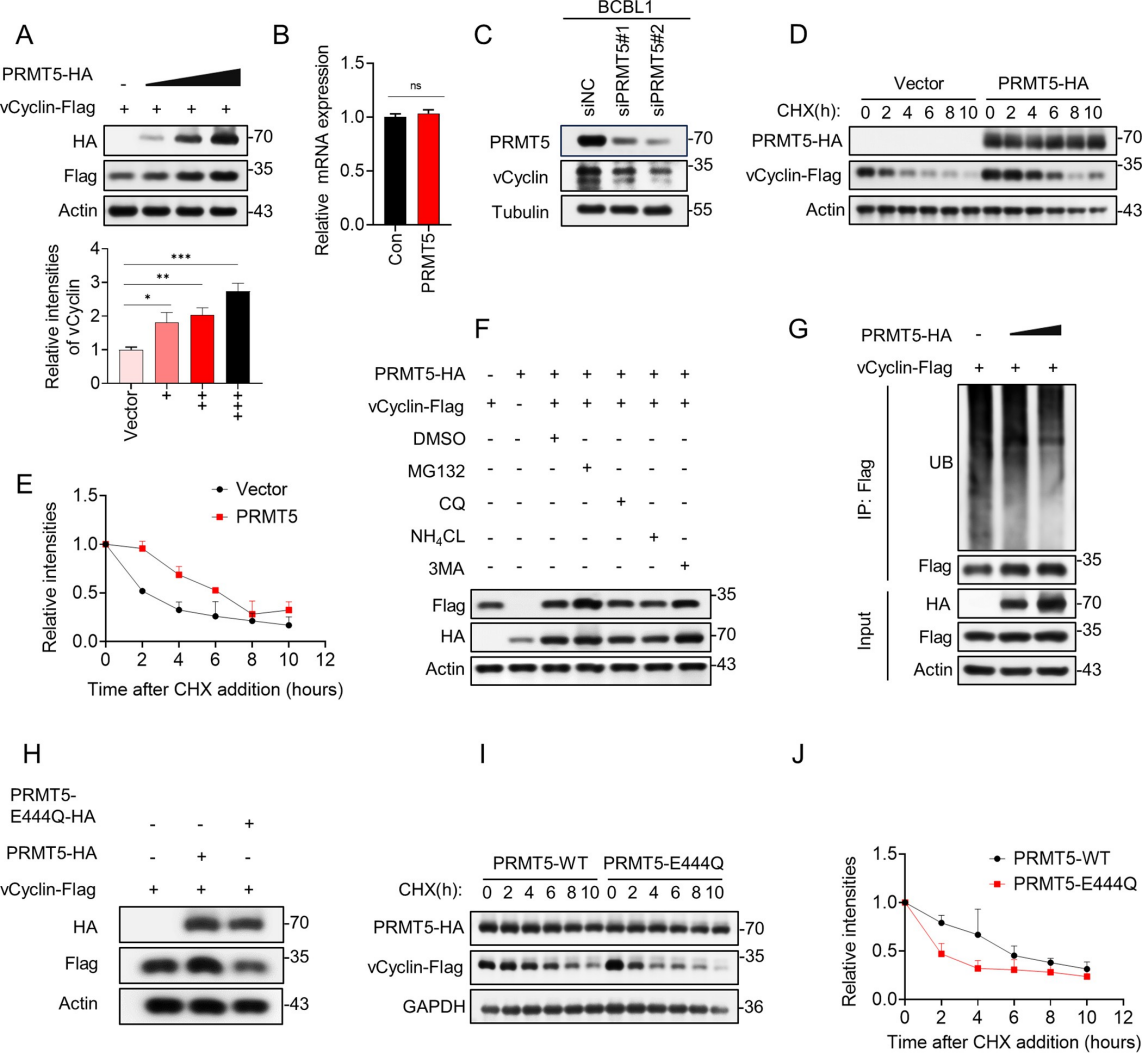
PRMT5 stabilizes vCyclin protein expression in a manner dependent on its methyltransferase activity. (A-B) Effect of PRMT5 on vCyclin expression. First, HEK293T cells were cotransfected with fixed amounts of vCyclin expression plasmid and increasing amounts of PRMT5 expression plasmids. The expression of vCyclin proteins were examined by immunoblotting (A) and qPCR (B). The relative band intensities of vCyclin from three biological replicates were graphically plotted at the bottom (A). (C) BCBL1 cells were transfected with two PRMT5 siRNAs. 72 h post-transfection, lysates of cells were subjected to western blot analysis with anti-PRMT5 and anti-vCyclin antibodies. (D) PRMT5 overexpression extended the half-life of vCyclin. HEK293T cells were transfected with Flag-tagged vCyclin with or without HA-tagged PRMT5 for 24h. Then, cells were treated with 100 μg/ml of CHX at different time points as indicated, then lysed and analyzed by immunoblotting with indicated antibodies. (E) The relative protein abundances of vCyclin from immunoblots (D) were quantified by band intensities. Representative results from three biological replicates are presented. (F) PRMT5 stabilized vCyclin protein expression by inhibiting proteasome-mediated degradation. HEK293T cells were transfected with Flag-vCyclin alone or HA-PRMT5 alone or Flag-vCyclin together with HA-PRMT5. 24h posttransfection, the cotransfected groups were treated with DMSO, MG132 (20μM), CQ (50μM), NH4CL (10mM) and 3MA (0.5mM) respectively for another 10h followed by detecting with western blots. (G) PRMT5 decreased vCyclin protein ubiquitination. HEK293T cells were transiently transfected with Flag-vCyclin alone or together with HA-PRMT5. After 24h transfection, the cells were treated with MG132 (20μM) for another 10h, followed by immunoprecipitated with anti-Flag antibody and then analyzed by immunoblotting with anti-ubiquitin antibodies. (H) PRMT5 stabilized vCyclin in an arginine methyltransferase activity-dependent manner. HEK293T cells were transfected with Flag-vCyclin alone or Flag-vCyclin together with HA-PRMT5 or Flag-vCyclin together with HA-PRMT5-E444Q. At 36 hours after transfection, WCLs were harvested and analyzed by immunoblotting. (I) HEK293T cells were co-transfected with Flag-vCyclin and HA-PRMT5 or HA-PRMT5-E444Q for 24h and then treated with CHX for the indicated times. (J) The graph shows the quantitative results from (I). Representative results from three biological replicates are presented.

The above findings suggested that PRMT5 can promote cell cycle progression and proliferation of KSHV latently infected cells. And PRMT5 can directly interact with vCyclin which is critical to the cell cycle and cell proliferation processes; thus, it is possible that this function of PRMT5 is due to its interaction with vCyclin. We therefore first tested whether PRMT5 affects KSHV vCyclin protein expression. HEK293T cells were cotransfected with increasing amounts of HA-tagged PRMT5 plasmids and fixed amounts of Flag-tagged vCyclin plasmids. The results showed that PRMT5 increased vCyclin protein expression in a dose-dependent manner (Fig 4A), while it had no impact on vCyclin mRNA expression (Fig 4B). The relative band intensities of vCyclin-Flag from three biological replicates were plotted graphically at the bottom of Fig 4A. In addition, we knocked down PRMT5 in BCBL1 cells and detected changes in endogenous vCyclin. As shown in Fig 4C, the expression of vCyclin decreased with the knockdown of PRMT5. To further investigate the role of PRMT5 in the stability of vCyclin, we measured the half-life of vCyclin in HEK293T cells using the protein synthesis inhibitor cycloheximide (CHX). Notably, coexpression of PRMT5 prolonged the half-life of vCyclin (Fig 4D and 4E).

Previously, PRMT5 has been suggested to alter protein stability by inhibiting protein ubiquitination [48]. In addition, studies have shown that both the autophagy–lysosome pathway and the ubiquitin–proteasome pathway are involved in eukaryotic intracellular degradation systems [49–52]. To further explore whether PRMT5 stabilizes vCyclin by inhibiting the proteasome or lysosomal pathways, we analyzed the ameliorative effect of the proteasome inhibitor MG132, the lysosome inhibitors NH_4_Cl and chloroquine (CQ) and the autophagy inhibitor 3-methyladenine (3-MA) on the degradation of vCyclin. As shown in Fig 4F, the proteasome inhibitor MG132 but not the lysosomal inhibitor CQ/NH_4_Cl or the autophagy inhibitor 3-MA significantly rescued vCyclin from degradation. To further evaluate the impact of PRMT5 on vCyclin ubiquitination, we coexpressed PRMT5 and vCyclin plasmids in HEK293T cells. After 24h transfection, the cells were treated with MG132 (20μM) for another 10h, and the cell lysate was immunoprecipitated with an anti-Flag antibody prior to immunoblotting with an anti-ubiquitin antibody. The results showed that ectopic PRMT5 expression led to a significant decrease in the ubiquitin level compared to that in the control group (Fig 4G).

To further define whether this regulation of vCyclin was related to the enzymatic activity of PRMT5, we constructed an enzyme activity-dead mutant, PRMT5-E444Q [53]. To test the hypothesis, we coexpressed Flag-vCyclin together with HA-PRMT5 or HA-PRMT5-E444Q in the experimental group and Flag-vCyclin together with HA vector in the control group. Our data showed that PRMT5-E444Q did not increase KSHV vCyclin protein expression unlike wild-type PRMT5 (PRMT5-WT) (Fig 4H). Consistent with the above results, PRMT5-E444Q resulted in a shorter half-life of vCyclin than PRMT5-WT (Fig 4I and 4J). We also investigated the impact of PRMT5 on the stability of cyclinD2 due to the high homology between vCyclin and cellular cyclinD2 (S4 Fig). In HEK293T cells, cotransfection of HA-PRMT5-WT or HA-PRMT5-E444Q with HA-cyclinD2 showed that PRMT5 does not upregulate the expression of cyclinD2 (S4A Fig). Furthermore, the half-life of cyclinD2 remains unaffected by PRMT5 (S4B and S4C Fig). More importantly, knockdown of PRMT5 resulted in decreased expression of vCyclin but not cyclinD2 (S4D Fig).

Together, our data suggest that PRMT5 significantly stabilizes vCyclin protein expression in a manner dependent on its enzymatic activity.

### vCyclin can be symmetrically di-methylated at arginine residues by PRMT5

PRMT5 is a mammalian protein arginine methyltransferase that catalyzes both histone and nonhistone reactions. Our previous results showed that the transcript levels of KSHV genes were not affected by knockdown of PRMT5, and that PRMT5 stabilized vCyclin protein expression. Therefore, we hypothesized that PRMT5 may catalyze the symmetric di-methylation of arginine (sDMA) on vCyclin protein.

**Fig 5:**
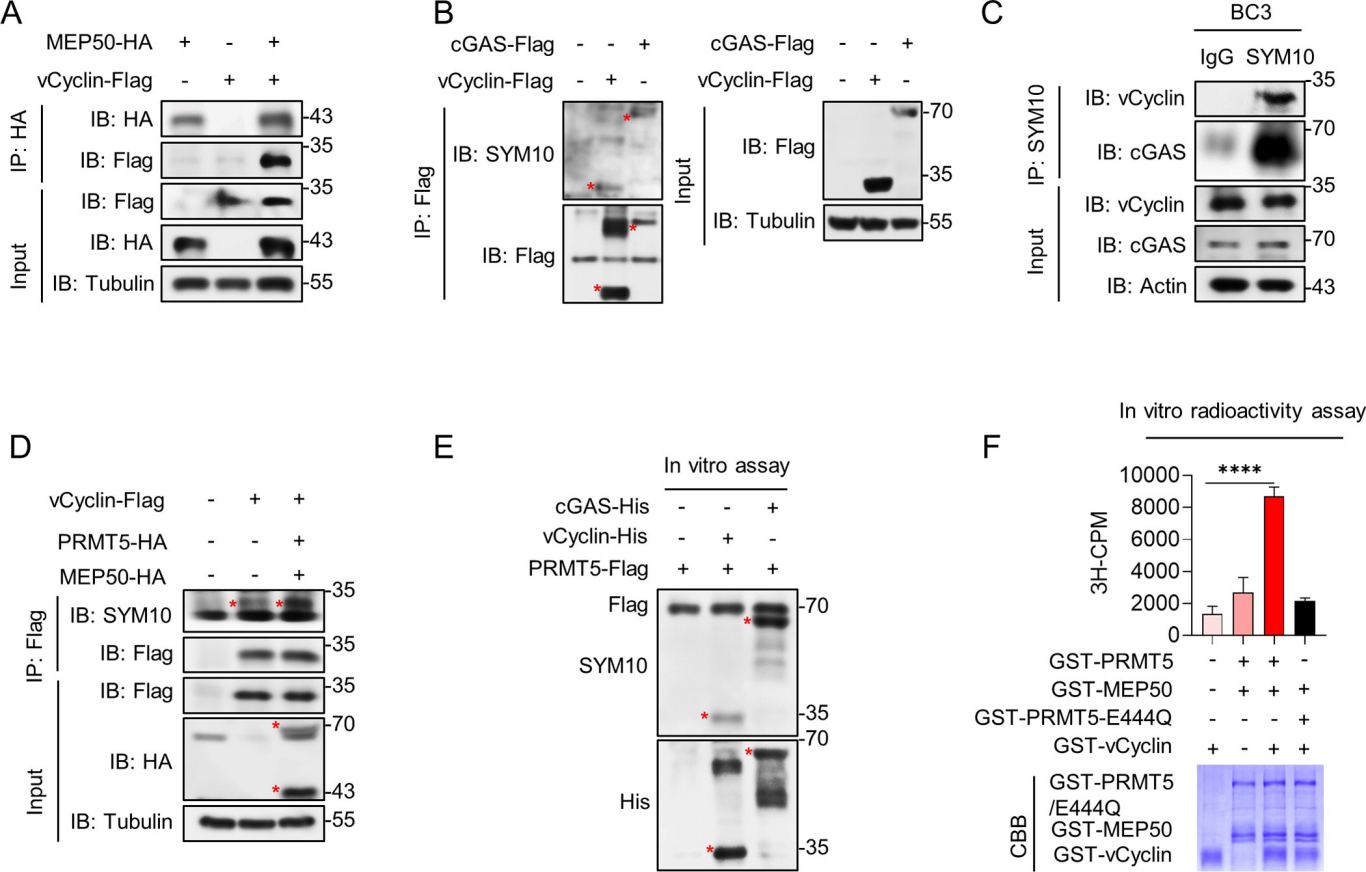
vCyclin can be symmetrically di-methylated at arginine residues by PRMT5. (A) co-IP analysis of the interaction between vCyclin and MEP50. HEK293T cells were cotransfected with Flag-vCyclin plasmids alone or HA-MEP50 plasmids alone or Flag-vCyclin and HA-MEP50 plasmids. IP assay was done by anti-HA antibodies to show the interaction between vCyclin and MEP50. (B) IP analysis of vCyclin dimethylation was done by transfecting Flag-vCyclin or Flag-cGAS in HEK293T cells, and then immunoblotted with anti-SYM10 antibodies. (C) Cell lysates from BC3 cells were immunoprecipitated with anti-SYM10 antibodies and then immunoblotted with KSHV anti-vCyclin antibodies and human anti-cGAS antibodies. (D) IP analysis of vCyclin dimethylation was done by transfecting Flag-vCyclin plamids alone or cotransfected with Flag-vCyclin and HA-PRMT5 plamids in HEK293T cells. Then the cells were lysed and immunoprecipitated with anti-Flag antibodies and then analyzed by immunoblotting with anti-SYM10 antibodies. Equal amounts of Flag and HA vectors were transfected into the first group as a negative control. (E) Immunoblot analysis of vCyclin dimethylation in an in vitro methylation system. Recombinant His-vCyclin/cGAS and the Flag-PRMT5 complex purified from HEK293T were incubated in methylation reaction buffer, and then the methylation of vCyclin was analyzed by immunoblotting with anti-SYM10 antibodies. (F) Liquid scintillation counting showing the methylation status of vCyclin by PRMT5 in the in vitro methylation system with H^3^-SAM (top). The indicated proteins used in the sequential reactions were examined by Coomassie blue staining (bottom). Representative results from three biological replicates are presented. Error bars indicate SD. Data were analyzed with Student’s multiple t-tests (****p< 0.0001).

PRMT5 methylates arginine residues, usually in a complex with its cofactor, methylosome protein 50 (MEP50) [54,55]. Thus, we first demonstrated the interaction between vCyclin and MEP50 by co-IP assay (Fig 5A). To further test whether PRMT5 could methylate vCyclin, we performed a series of experiments both in vitro and in vivo. We transfected Flag-tagged vCyclin plasmids and Flag-tagged cGAS plasmids into HEK293T cells. Since cGAS has been demonstrated to be methylated by PRMT5 [56], the cGAS group was utilized as a positive control in this instance. Then, we measured the sDMA modification status of vCyclin via western blotting using an antibody against symmetrically di-methylated arginine (SYM10). The IP assay indicated that vCyclin and cGAS could be symmetrically di-methylated (Fig 5B). As expected, the modification of vCyclin was further confirmed via IP using an anti-SYM10 antibody and via immunoblotting (IB) with KSHV anti-vCyclin antibody and human anti-cGAS antibody in BC3 cells (Fig 5C). Moreover, exogenous overexpression of PRMT5 and MEP50 increased the sDMA of vCyclin (Fig 5D). Furthermore, we verified that vCyclin could be methylated by the PRMT5-MEP50 complex in an in vitro methylation system, which was conducted with recombinant His-vCyclin/cGAS and the Flag-PRMT5 complex purified from HEK293T (Fig 5E). Consistently, an in vitro radioactivity assay was conducted with recombinant GST proteins. GST recombinant proteins as shown in Fig 5F were incubated with H^3^- S-adenosylmethionine (SAM) in methylation buffer. Then, we measured the total ^3^H-SAM incorporation by liquid scintillation counting. It turned out that vCyclin was directly methylated by PRMT5 with a substantial increase in H^3^-SAM binding. Additionally, we conducted another in vitro methylation assay, which demonstrated that PRMT5 is unable to catalyze the sDMA of cyclin D2 in vitro (S4E Fig).

### PRMT5-mediated vCyclin dimethylation occurs at arginine 128 (Arg128)

**Fig 6:**
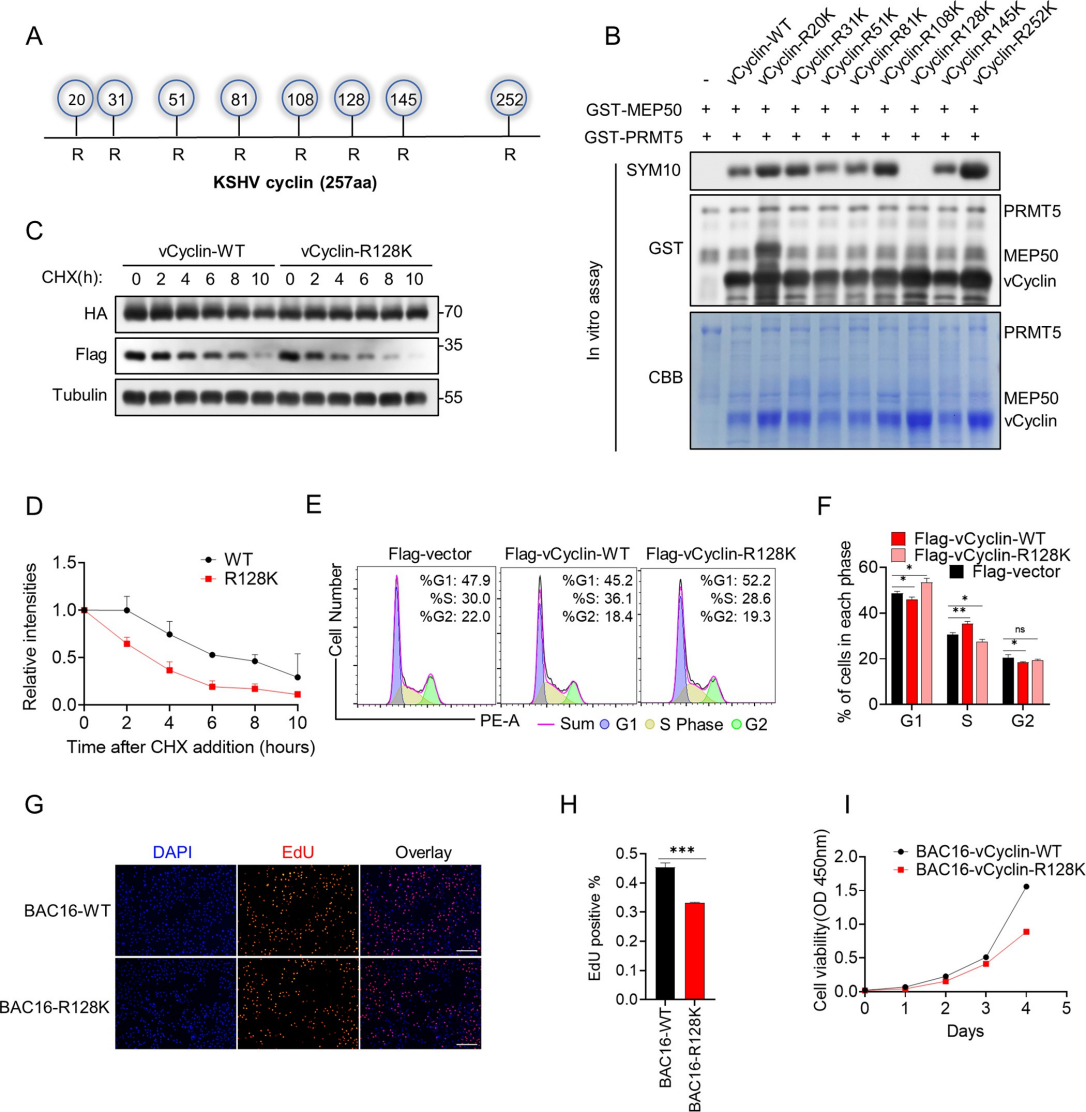
PRMT5-mediated vCyclin dimethylation occurs at arginine 128. (A) Schematic diagram of all arginine sites on the vCyclin protein sequence. (B) Validation of Arg128 as the vCyclin methylation site by PRMT5 in the in vitro system. Purified GST-vCyclin mutants together with GST-PRMT5, GST-MEP50 and SAM were subjected to in vitro methylation buffer to detect vCyclin methylation. Immunoblotting (two top panels) and Coomassie blue staining (bottom panel) was shown. (C) HEK293T cells were co-transfected with HA-PRMT5 and Flag-vCyclin-WT or Flag-vCyclin-R128K for 24 h and then cells were treated with CHX at different time points as indicated, then lysed and analyzed by immunoblotting with indicated antibodies. (D) The graph displays the quantitative results obtained from (C). (E-F) HEK293T cells were transfected separately with Flag-vector, Flag-vCyclin or Flag-vCyclin-R128K for 30 h, and then flow cytometry was performed to analyze the cell cycle distributions. Representative results from three biological replicates are presented. Error bars indicate SD. Data were analyzed with Student’s multiple t-tests (*p < 0.05, **p< 0.01). (G-I) Flow cytometry-sorted BAC16-WT and BAC16-vCyclin-R128K cells were counted and plated for EdU and CCK8 assays. For CCK8 assays (I), 2 × 10^3^ cells were seeded into 96-well plates, After the cells adhered, CCK8 reagent was added on day 1, 2, 3, 4 and the absorbance was measured after 1 hours. For EdU assays (G-H), 2 × 10^5^ cells were seeded to coverslips and then treated with 10μm EDU for 2h. Representative immunofluorescence images show the expression of EdU. Scale bars represent 50μm. Representative results from three biological replicates are presented. Error bars indicate SD. Data were analyzed with Student’s multiple t-tests (***p< 0.001).

Upon analyzing the sequence of vCyclin, we discovered that it contains 8 arginine residues (Fig 6A). To determine the modification sites, in vitro arginine methylation assays were performed using recombinant GST-mutants and GST-PRMT5/MEP50.

The results demonstrated that the substitution of Arg128 with lysine (R128K) almost completely abolished the symmetrical di-methylation of vCyclin induced by PRMT5 (Fig 6B), indicating that the Arg128 residue is the PRMT5 potential catalytic site.

To validate whether the Arg128 residue is required for regulation of the stability of vCyclin induced by PRMT5, we determined the effect of PRMT5 on the half-lives of vCyclin-WT and vCyclin-R128K. HEK293T cells were co-transfected with HA-PRMT5 and Flag-vCyclin or Flag-vCyclin-R128K for 24 h and then cells were treated with CHX at different time points as indicated. Notably, the half-life of vCyclin-R128K was significantly shortened by compared to that of vCyclin-WT (Fig 6C and 6D). In order to exclude the impact of other arginine residues on the stability of vCyclin, we randomly chose vCyclin-R51K to conduct a consistent half-life assay as described above. The findings indicated that PRMT5 did not affect the half-life of vCyclin-R51K compared to the vCyclin-WT (S5 Fig).

Based on the previous data in Fig 4, we speculated that methylation at Arg128 of vCyclin might affect the cell cycle progression and cell proliferation. To test this hypothesis, HEK293T cells were separately transfected with plasmids encoding Flag-vector, Flag-vCyclin-WT and Flag-vCyclin-R128K. Flow cytometry analysis showed that overexpression of vCyclin-WT promotes the G1 to S transition, while overexpression of vCyclin-R128 abolished the increase observed in the vCyclin-WT group (Fig 6E and 6F). To investigate the effect of KSHV vCyclin Arg128 residue on cell proliferation, we constructed a recombinant KSHV in which Arg128 residue in vCyclin was mutant by bacterial artificial chromosome (BAC) technology. Then we established cell lines infected with this recombinant KSHV (BAC16-R128K) and KSHV BAC16-WT as previously reported [57] (S6 Fig). Sanger sequencing showed that BAC16-R128K point mutant plasmid was successfully constructed (S6B Fig). Then BAC16-R128K and BAC16-WT plasmids were transfected into iSLK cells and the positive cells (green fluorescence) were sorted by flow cytometry (S6C and S6D Fig). We also assessed their ability to produce viral progeny and to infect new cells. Equal numbers of BAC16-WT and BAC16-R128K live cells were plated and inducted with doxycycline (Dox) and sodium butyrate (NaB). Then the viral supernatant was used to infected HEK293T cells. At 36 h post-infection, HEK293T cells were collected for detecting the relative DNA levels of KSHV (S6E Fig). After that, BAC16-WT and BAC16-R128K cells were plated for the EdU and CCK8 proliferation assays after determining viable cell counts (Fig 6G–6I). CCK8 (Fig 6I) and EdU (Fig 6G and 6H) cell proliferation assays revealed that cell proliferation was slower in BAC16-R128K cells than in BAC16-WT cells.

The above results suggest that di-methylation of vCyclin at Arg128 residue by PRMT5 is crucial for the stabilization of vCyclin and regulation of cell cycle progression and cell proliferation.

### The methylation of vCyclin by PRMT5 increases the phosphorylation of RB

**Fig 7:**
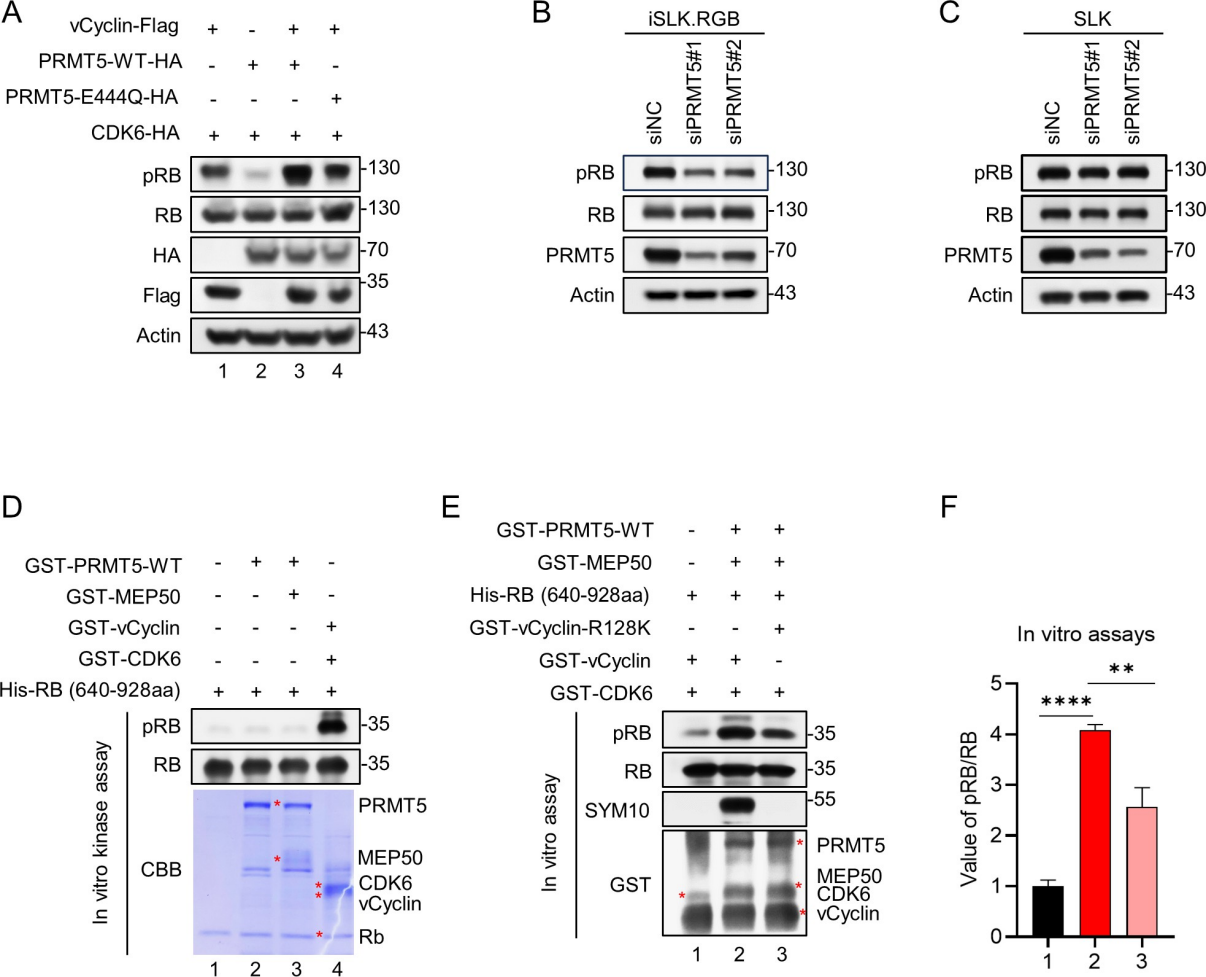
The methylation of vCyclin by PRMT5 increases the phosphorylation of RB. (A) HEK293T cells were transfected with PRMT5 (lane 3) or PRMT5-E444Q (lane 4) or without PRMT5 (lane 1) and other plasmids as indicated. At 48 hours after transfection, WCLs were harvested. Both the phosphorylation of RB and the expression of RB was analyzed by immunoblotting. (B) Knockdown of endogenous PRMT5 reduced the phosphorylation of RB. iSLK.RGB cells were transfected with two PRMT5 siRNAs. 72 h post-transfection, lysates of cells were subjected to western blot analysis. (C) PRMT5 knockdown in SLK cells had no impact on the phosphorylation or expression of RB. (D) PRMT5 and MEP50 had no effect on the phosphorylation of RB. Purified RB alone, RB and PRMT5, RB and PRMT5/MEP50 were subjected to in vitro kinase analysis by immunoblotting with anti-pRB antibodies. The vCyclin and CDK6 group (lane 4) was as positive control. (E) The methylation of vCyclin by PRMT5 enhances its function in the phosphorylation of RB. Purified vCyclin/vCyclin-R128K, PRMT5/MEP50 proteins were added into methylation reaction buffer. Subsequently purified RB and CKD6 proteins were added into kinase reaction buffer. Then the reaction buffer was subjected to SDS-PAGE and immunoblotted with the indicated antibodies. (F) The relative protein abundances of pRB/RB from immunoblots (E) were quantified by band intensities. Representative results from three biological replicates are presented. Error bars indicate SD. Data were analyzed with Student’s multiple t-tests (**p< 0.01, ****p< 0.0001).

Previous studies have shown that CDK6 can be stimulated by vCyclin to phosphorylate the RB protein [11]. Hence, we speculated that the methylation of vCyclin by PRMT5 may affect pRB signaling to regulate the cell cycle. To test this hypothesis, we first assessed the phosphorylation level of the RB protein in HEK293T cells after transfecting the cells with the indicated plasmids. Interestingly, the phosphorylation of RB increased in the presence of PRMT5 (Fig 7A, lane 3 vs. lane 1), whereas it decreased in the presence of PRMT5-E444Q (Fig 7A, lane 4 vs. lane 3). However, PRMT5 had no effect on the expression level of RB itself. Next, to corroborate the above findings, we knocked down PRMT5 in endogenous iSLK.RGB cells, which significantly decreased the phosphorylation level of RB but did not affect the expression of RB itself (Fig 7B). Importantly, PRMT5 knockdown in SLK cells had no discernible impact on the phosphorylation or expression of RB (Fig 7C). To exclude a direct influence of PRMT5 on RB phosphorylation, we designed in vitro phosphorylation experiments (Fig 7D). The results showed that the presence of PRMT5 alone or in combination with MEP50 did not affect the phosphorylation of RB. The vCyclin and CDK6 group (lane 4) was as positive control. To further verify that the sDMA of vCyclin induced by PRMT5 was responsible for the phosphorylation of RB, we conducted the sequential in vitro methylation/phosphorylation assays. We first performed in vitro methyltransferase assays in methylation reaction buffer with purified vCyclin/vCyclin-R128K, PRMT5 and MEP50 proteins, and then subsequently added purified RB, CKD6, and kinase reaction buffer at 30°C for 2 hours. As expected, the phosphorylation level of RB was elevated when vCyclin was methylated (Fig 7E, lane 2 vs. lane 1), while it was decreased in vCyclin-R128K group (Fig 7E, lane 3 vs. lane 2). The relative band intensities of pRB/RB from three biological replicates were represented in Fig 7F.

Together, these data indicate that PRMT5 promotes vCyclin/CDK6-mediated phosphorylation of RB via di-methylation of vCyclin.

## Discussion

**Fig 8:**
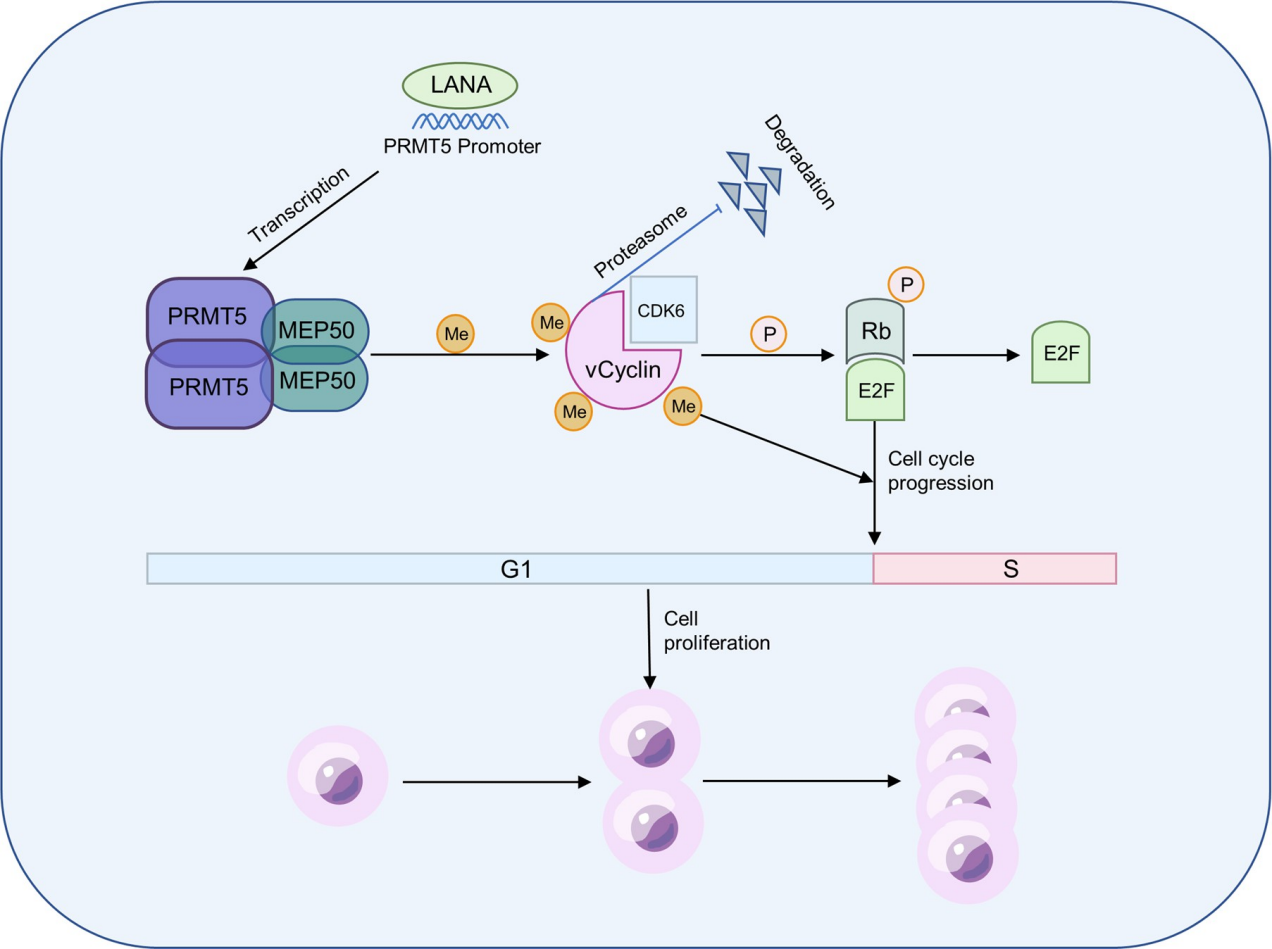
Working model for the effect of PRMT5 on vCyclin in regulating cell cycle progression and cell proliferation in KSHV-infected cells. During KSHV latency, the host protein PRMT5 is upregulated by LANA through transcriptional activation. PPRMT5 further interacts with KSHV vCyclin and dimethylates vCyclin symmetrically at arginine 128 to stabilize vCyclin, thereby promoting the cell cycle and cell proliferation of KSHV-infected cells by increasing the phosphorylation of RB.

KSHV, as a human oncogenic DNA virus, can initiate lymphoproliferative diseases and malignancies [1]. However, the mechanisms underlying KSHV’s promotion of tumorigenesis and development remain inadequately understood. D-type cyclins are central cell cycle regulators and are among the most frequently dysregulated therapeutic targets in human malignancies [58]. vCyclin encoded by KSHV ORF72 is the homolog of cellular D-cyclin, which regulates virus replication and cell proliferation by constitutively activating the CDK-RB pathway [59]. Thus, understanding the processes and PTMs that regulate vCyclin is crucial for preventing and treating viral cancers. Our study found a new vCyclin binding host factor, PRMT5, which not only methylates vCyclin to promote cell cycle progression and cell proliferation but also promotes KSHV latency. Mechanistically, PRMT5 catalyzes symmetrical dimethylation of vCyclin at Arg 128, thereby impacting the pRB/E2F pathway. In summary, our data provide a working model that elucidates the involvement of PRMT5 in the control of vCyclin and in the cell cycle and proliferation of cells with latent KSHV infection (Fig 8).

Recent studies have linked elevated PRMT5 levels to a variety of human disorders, particularly cancers, including lung cancer, breast cancer, leukemia, lymphoma, gastric cancer, and colorectal cancer [60, 61]. The regulation of proliferation by PRMT5 and the direct interaction of PRMT5 with proteins that are frequently dysregulated or mutated in cancer indicate that PRMT5 functions as an oncogene [62]. Multiple transcription factors regulate PRMT5 expression, such as Myc and NF-ΚB, which can upregulate PRMT5 in numerous B cells [63]. Similarly, our findings are in accord with recent studies indicating that LANA can upregulate PRMT5 through transcriptional activation in KSHV latent cells (Fig 2). These results indicate that PRMT5 may play a role in KSHV-related malignancies. Given the current absence of accessible strategies to target PRMT5, it is imperative to investigate PRMT5 inhibitors.

It has been reported that PRMT5 methylates the Epstein–Barr virus (EB) nuclear antigen (EBNA) protein and stimulates EBNA-dependent transcription, suggesting that PRMT5 may also play a role in latent EB infection [64, 65]. Unexpectedly, another study has shown that PRMT1 but not PRMT5 directs the methylation of LANA in KSHV [66]. In addition, reductions in PRMT5 levels results in a decline in symmetric methylation on H4R3 and an elevation in viral gene transcription, validating the inhibitory function of PRMT5 in KSHV reactivation [67]. However, the role of PRMT5 in KSHV latent cells remains poorly understood. Our study reveals that PRMT5 can methylate and stabilize the KSHV encoded oncogene vCyclin, which promotes cell cycle progression and cell proliferation in KSHV latent cells (Fig 3). Meanwhile, numerous recent articles have documented the antiviral properties of PRMT5. For example, the PRMT5/WDR77 complex inhibits the replication of the hepatitis E virus by catalyzing the methylation of ORF1 to impair the replicase activity of the virus [68]. Through epigenetic repression of covalently closed circular DNA transcription and disruption with pregenomic RNA encapsidation, PRMT5 inhibits hepatitis B virus replication [69]. The methylation of cGAS by PRMT5 attenuates the cGAS-mediated antiviral immune response by inhibiting cGAS DNA binding [56]. These findings provide additional insight into the role of PRMT5 as an oncogene involved in the latency of oncogenic viruses but also as an antiviral protein involved in virus replication. Previous studies and our present study indicate that PRMT5 is a promising therapeutic target for virus-associated tumors.

D-type cell cycle proteins are fundamental regulators of the cell cycle and some of the most prevalent therapeutic targets in the treatment of human cancer [58]. Recent studies on the regulatory mechanisms of ubiquitination of D-type cyclins have been extensive [70–72]. The seven substrate receptors in the CUL1-RING ubiquitin ligase complex, FBXO4, FBXO31, β-TrCP, FBXW8, SKP2, FBXL2, and FBXL8, as well as the APC/C (anaphase-promoting complex/cyclosome) complex, have been suggested to engage in targeted degradation of D-type cyclins [73]. In our research, we demonstrated that PRMT5 stabilizes vCyclin by inhibiting the ubiquitin–proteasome degradation pathway (Fig 4D–4F). Next, we intend to further investigate the E3 ubiquitin ligases targeting vCyclin through tandem affinity purification/mass spectrometry detection and bioinformatic analysis. On the other hand, some groups have reported that there is a possible crosstalk between PRMT5-mediated methylation and E3 ligase-driven linear ubiquitination [48,74]. In this study, we investigated whether PRMT5 efficiently dimethylates vCyclin at theArg128 residue (Fig 6). Next, we will further explore the ubiquitination site of vCyclin and whether the methylation site of vCyclin engages in cross-talk with the ubiquitination site of vCyclin. In general, there is currently almost no research on the PTMs (methylation or ubiquitination) of vCyclin. Thus, more investigation and discovery are needed.

KSHV vCycin is a homolog of cellular cyclin D2 with a 29.8% sequence homology [17]. However, vCyclin exhibits greater stability, a wider array of functions in the cell cycle, and a longer length of activity compared to cellular cyclin D2. Prior research has demonstrated that vCyclin has a longer half-life (~6 hours) than cyclin D2 (~30 minutes). According to this study, the instability of cyclin D2 is caused by the PEST motif [33]. In our investigation, we discovered a novel mechanism by which PRMT5 methylates vCyclin but not cyclinD2, thereby stabilizing vCyclin (Fig 4). Then we compare the impact of PRMT5 and PEST motif on vCyclin stability. We created a hybrid protein called vCyclin-PEST by combining the whole 257-amino acid sequence of vCyclin with the last 27 amino acids of cellular cyclin D2, which contain a PEST motif (S7A Fig). Then, HEK293T cells were co-transfected with HA-PRMT5 and Flag-vCyclin or Flag-vCyclin-PEST for 24 h and then cells were treated with CHX at different time points as indicated. The findings indicated that PRMT5 significantly prolonged the half-life of vCyclin to 6 hours. Nevertheless, even with PRMT5 overexpression, the half-life of vCyclin significantly decreased when it was combined with the PEST sequence (S7B Fig). These findings suggest that the PEST sequence plays a primary role in the different stability between vCyclin and cyclin D2.

Previously, numerous studies have shown that strong correlations exist between the PRMT5/MEP50 complex and the cyclin/CDK complex, and interactions between these complexes are always possible. The aggarwal group ascertained that cyclin D1T286A, CDK4, MEP50 and PRMT5 coprecipitate in B cells and human cancer cells. They also showed that cyclinD1/CDK4 phosphorylate MEP50 to increase PRMT5/MEP50 activity [28]. Huang’s group showed that competitive inhibition of the interaction between CDK4 and CDKN2A by PRMT5 is essential for glucose-induced hepatocellular carcinoma cell proliferation [75]. PRMT5 is a potential oncoprotein that upregulates G1 cyclins/cyclin-dependent kinase [76]. Our data also corroborate the above conclusions of the existence of a strong interaction between PRMT5/MEP50 and vCyclin/CDK (Figs 1 and 5A and S4 Fig). Given these findings, the crystal structure of the PRMT5/MEP50 complex and Cyclin/CDK complex needs to be analyzed. Research on this crystal structure will constitute a promising new research field and help to thoroughly decipher the mechanism of the fine-tuned modulation of the cell cycle and cell proliferation pathway.

In conclusion, our research revealed for the first time that PRMT5 is a new vCyclin-binding protein and that PRMT5 symmetrically methylates vCyclin, thereby promoting KSHV latency. Given the essential role of PRMT5 in KSHV latency, inhibiting PRMT5 expression in KSHV-infected cells could be an effective therapeutic strategy for KSHV-related diseases.

## Materials and methods

### Cell culture

The MM, KMM [77], SLK, iSLK.RGB [78], iSLK, HEK293T, HEK293T-KSHV, and Hela cell lines were cultured in DMEM (HyClone) supplemented with 10% FBS (Biological Industries), antibiotics (penicillin and streptomycin, Gibco). iSLK.RGB (10μg/ml puromycin, Sigma; 250μg/ml G418, Sigma; and 250μg/ml hygromycin B, Roche) and KMM (150μg/ml hygromycin B) cells were cultured under the appropriate selection conditions. KSHV-positive B lymphoma cell lines (BCBL1, BJAB-KSHV) and KSHV-negative B lymphoma cell lines (BJAB) were cultured in RPMI 1640 (HyClone) supplemented with 10% FBS (HyClone) and 1% antibiotics (penicillin and streptomycin, Gibco). All the cell lines were grown at 37°C in a humidified environment supplemented with 5% CO2.

### Antibodies and reagents

The following primary antibodies were used: anti-PRMT5 rabbit monoclonal (ABclonal, A19533), anti-vCyclin rat monoclonal (Abcam, ab12208), anti-LANA rat monoclonal antibody (Advanced Biotechnology Inc, 13-210-1000), anti-GST (ABclonal, AE001) anti-His (ABclonal, AE003), anti-ubiquitin antibody (ABclonal, A3207), anti-Flag antibody (Sigma, F1804), anti-HA (Sigma-Aldrich, H6908), anti-α-Tubulin antibody (Sigma-T6199), anti-GAPDH (Sigma-Aldrich, G8795), anti-β-actin rabbit monoclonal antibody (ABclonal, AC026), anti-SYM10 (Sigma, 07–412), anti-pRB (abcam, ab173289), anti-RB (abcam, ab ab181616), anti-cGAS (CST, #15102), anti-cyclinD2 (abcam, ab308258). The secondary antibodies used in western blotting and immunofluorescence assays were HRP-conjugated anti-mouse or anti-rabbit IgG (Jackson ImmunoResearch Laboratories), HRP-conjugated anti-rat IgG (ABclonal, AS028) and goat anti-rat antibodies conjugated with Alexa Fluor 568 (Thermo Fisher Scientific, A-11077) and goat anti-rabbit antibodies conjugated with Alexa Fluor 647 (Thermo Fisher Scientific, A-21244).

The other reagents used (and their sources) were as follows: anti-FLAG M2 affinity gel (Sigma, A2220), recombinant protein A agarose (Invitrogen, 15948–014), recombinant protein G agarose (Invitrogen, 15920–010), glutathione Sepharose 4B (GE Healthcare, 17-0756-01), the TNT T7 Quick Coupled transcription/translation system (Promega L1171). MG132 (MedChemExpress, HY-13259), cycloheximide (CHX) (MedChemExpress, HY-12320), 3-Methyladenine (3MA) (MedChemExpress, HY-19312), chloroquine (MedChemExpress, HY-17589A), NH4Cl (Sigma, A9434), protease inhibitor cocktail (Sigma, P8340), EPZ015666/GSK3235025 (Selleck, S7748), H^3^-SAM (Perkinelmer, NET155V250UC), Dox and NaB were purchased from Sigma-Aldrich (St. Louis, MO)

### Plasmids

PRMT5 gene was amplified from an iSLK.RGB cell cDNA library. MEP50, CDK6, RB and cyclinD2 gene were amplified from an HEK293T cell cDNA library. The vCyclin coding sequence was amplified from KSHV BAC16 genomic DNA. The plasmids pCDH-SF-LANA and GST-LANA were described previously [79]. The plasmids pCDNA3.1-HA-cGAS were described previously [80]. The following plasmids were generated by PCR amplification and subcloned into the pCDH-Flag vector at EcoRI and BamHI sites: pCDH-Flag-vCyclin, pCDH-Flag-PRMT5, pCDH-Flag-LANA, pCDH-Flag-vCyclin-mutants. The following plasmids were produced by PCR amplification and subcloned into the pCDNA3.1-HA vector at EcoRI and XbaI sites: pCDNA3.1-HA-PRMT5, pCDNA3.1-HA-PRMT5-E444Q, pCMV-HA-vCyclin, pCMV-HA-MEP50, pCMV-HA-CDK6, pCMV-HA-cyclinD2. The following plasmids were produced by PCR amplification and subcloned into the pGEX-4T vector at EcoRI and XhoI sites: GST-vCyclin, GST-vCyclin-mutans, GST-PRMT5, GST-PRMT5-E444Q, GST-MEP50, GST-CDK6. His-RB was produced by PCR amplification and subcloned into the pET-30a vector at EcoRI and XhoI sites. The luciferase reporter plasmid pGL3-Enhancer-pPRMT5 was constructed by cloning the promoter regions of PRMT5 (-2000 to -1 bp) from the iSLK.RGB genomic library into the pGL3-Enhancer vector. All the primers used for PCR amplification are listed in the S4 Table.

### Mass spectrometry

The procedures were as described previously [57, 79]. Briefly, Flag-tagged vCyclin or the empty vector was overexpressed in HEK293T and HEK293T.219 cells. After 48 hours, the cells were rinsed thrice with pre-chilled phosphate-buffered saline (PBS) and lysed in ice-cold radioimmunoprecipitation assay IP lysis buffer (Beyotime Biotechnology, P0013) with protease inhibitor cocktail. The extract was loaded into anti-FLAG M2 agarose beads and then incubated with rotation for 6–8 h at 4°C, followed by washing with PBS buffer 4 times and monitored by SDS-PAGE and visualized with silver.

The protein identification via mass spectrometry (MS) was performed by SpecAlly Life Technology Co., Ltd, Wuhan, China. The procedure and analysis methods were described briefly. All samples were analyzed on timsTOF Pro (Bruker Daltonics), a hybrid trapped ion mobility spectrometer (TIMS) quadrupole time-of-flight mass spectrometer. An UltiMate 3000 RSLCnano system (Thermo) was coupled to timsTOF Pro with a CaptiveSpray nano ion source (Bruker Daltonics). Peptide samples were injected into a C18 Trap column (75 μm*2 cm, 3 μm particle size, 100 Å pore size, Thermo), and separated in a reversed-phase C18 analytical column (75 μm*15 cm, 1.7 μm particle size, 100 Å pore size, IonOpticks). Mobile phase A (0.1% formic acid in water) and mobile phase B (0.1% formic acid in ACN) were used to establish the seperation gradient at a flow rate of 300 nL/min. The MS data acquisition was performed in PASEF mode. The capillary voltage was set to 1500 V. The MS and MS/MS spectra were acquired from 100 to 1700 m/z. The ion mobility was scanned from 0.75 to 1.4 Vs/cm2. The accumulation time and ramp time were set to 100 ms. The acquisition cycle of 1.16 s comprised one full MS scan and 10 PASEF MS/MS scans. MS raw data were analyzed with MaxQuant (V2.2.0.0) using the Andromeda database search algorithm. Spectra files were searched against the Human_gammaherpesvirus_8 protein sequence database (2023-01-23, 86 entries) from NCBI and the Human protein sequence database (2023-06-19, 20423 entries) from Uniprot. Further bioinformatics analysis was conducted in R statistical programming environment.

### Western blot and immunoprecipitation

Transfected cells were lysed for 30 minutes on ice in Western and IP lysis buffer (Beyotime Biotechnology, P0013) supplemented with 1 mM PMSF and a protease inhibitor cocktail after 48 hours post-transfection. Cell debris was removed by centrifugation at 12,000g for 15 minutes at 4°C, and 10% of the lysates were preserved for input control. The remaining lysate was immunoprecipitated using anti-Flag M2 affinity gel by rotating overnight at 4°C. The beads were pelleted and rinsed three times in lysis buffer then boiled in SDS loading buffer for Western blotting (WB) analysis. For WB, protein samples were analyzed through SDS-PAGE and transferred to nitrocellulose membranes (Bio-Rad Laboratories), followed by treating with the relevant antibodies, detecting with HRP-linked secondary antibodies, and visualizing using ECL reagents (GE).

### Protein purification and in vitro binding assay (GST pull-down)

The procedures were performed as described previously [79, 81]. Briefly, GST or GST fusion proteins were expressed in Escherichia coli strain BL21 (DE3), then inducted overnight at 16°C with isopropyl thiogalactopyranoside (IPTG). The protein was purified using a GST-tag Protein Purification Kit (Beyotime Biotechnology, P2262) according to the manufacturer’s instructions. In vitro translated PRMT5 was produced by the TNT-coupled transcription/translation system according to the manufacturer’s instructions. Then, the GST or GST fusion proteins and in vitro translated PRMT5 were incubated with GST-fusion-protein-bound beads overnight. After washing with RIPA buffer four times, the pull-down products were analyzed by western blotting.

### Immunofluorescence assay

iSLK.BAC16 cells were embedded with coverslips. When the cells reached the appropriate density, cells were washed by PBS and then fixed with 4% paraformaldehyde (Beyotime Biotechnology, P0099) for 15 min and permeabilized with 0.2% Triton X-100 for 10 min and blocked with 5% bovine serum albumin. After blocking with 5% bovine serum albumin (Life Technologies) in PBS for 1 h, cells were incubated with specific primary antibodies (1:50–1:200 dilution) overnight at 4°C, and further staining with the secondary antibodies (1:400 dilution) for 1 h at room temperature. Cell nuclei were stained with DAPI (Beyotime, C1002) for 10 min. Finally, the stained cells were visualized and captured by Leica SP8 laser confocal microscopy.

### Quantitative real-time PCR (RT-qPCR)

To determine the RNA levels or genomic DNA levels, quantitative real-time PCR was used. To analyze RNA levels, the total RNA was extracted using TRIzol reagent (Invitrogen) according to the manufacturer’s instructions. To analyze DNA levels, total DNA was extracted from the cells with the Genomic DNA Extraction Kit (Tiangen). Relative KSHV episomal copy numbers were calculated by qPCR amplification of the LANA and vCyclin as previously described [79, 82]. One microgram of RNA was reverse transcribed into cDNA using HiScript III RT SuperMix (Vazyme, R323-01). Subsequently, RT-qPCR was was performed with Hieff qPCR SYBR Green Master Mix (YEASEN, 11202ES03) on QuantStudio 6 Flex Real-Time PCR System (Applied Biosystems), following the manufacturer’s protocol. Relative mRNA levels and relative DNA levels were normalized to actin and calculated by the ΔΔCT method. The samples were tested in triplicate. The primers used in RT-qPCR are listed in the S5 Table.

### Dual-luciferase reporter assay

As directed by the manufacturer, the dual-luciferase reporter assay system (Promega) was employed. HEK293T cells were placed into 12-well plates at the proper density and then cotransfected the corresponding luciferase reporter plasmids with the Flag-tagged LANA plasmid or empty vector plasmid. Using Renilla luciferase activities as a reference, Firefly luciferase activities were normalized to the vector group as 1.0, taking Renilla luciferase activities as references.

### RNA interference

Cells were transfected with negative control siRNA and two siRNAs corresponding to the indicated genes (GenePharma Technology) using InvitroRNA (IVG1101-10) according to the manufacturer’s instructions. After 72 h post-transfection, the cells were harvested, and the efficiency of RNA interference was detected by immunoblotting analysis. All siRNA used in our experiments and their sequences are as follows:

negative control siRNA, 5’-UUCUCCGAACGUGUCACGUTT-3’;

siPRMT5#1, 5’-GGAUAAAGCUGUAUGCUGUUU-3’;

siPRMT5#2, 5’-CCCAGAAGAGGAGAAGGAUAUU-3’;

siLANA#1, 5’-AACUGUCCUUAUGGCUUUGTT-3’;

siLANA#2, 5’- GCUAGGCCACAACACAUCUTT-3’;

### Cell cycle analysis

The treated cells were collected and fixed overnight at -20°C with 75% pre-chilled ethanol. After removing the ethanol, the cells were washed twice with PBS buffer and then incubated for 30 minutes at room temperature with propidium iodide staining solution from the cell cycle analysis reagent (Beyotime Biotechnology, C1052). The cell cycle was analyzed by flow cytometry (Beckman CytoFlex), and the data was analyzed by Flowjo software.

### Cell proliferation assay

The cell proliferation was detected by EdU cell proliferation assay (Abbkine, KTA2030) and cell counting kit-8 assay (CCK-8) (Yeasen, 40203ES60) according to the manufacturer’s instructions. For the EdU cell proliferation assay, live cells were counted by automated cell counter (BioRad, TC20) with Trypan blue staining. Then 2 × 10^5^ cells were seeded to coverslips and treated with 10μm EDU for 2h. After incubation, the culture medium was removed, and then the cells were fixed, washed, and penetrated. Finally, 200ul Click-iT reaction mixture was added to incubate for 30min away from light. The samples were analyzed with a Leica fluorescence microscope. For the CCK-8 assay, 2 × 10^3^ cells/well were seeded into 96-well plates. After the cells adhered, 10 ul of CCK8 reagent was added to each well on days 1, 2, 3, 4 and 5, and the absorbance was measured by spectrophotometry at 450 nm wavelength after 1 hours.

### Construction and identification of KSHV BAC16-vCyclin-R128K mutant

The mutagenesis of KSHV BAC16-vCyclin-R128K coding sequence was modified using a two-step ‘scarless’ homologous recombination procedure that has been previously described [57, 83, 84]. Briefly, a linear DNA fragment was generated by PCR amplification using the pEPKan-S plasmid as a template. The PCR primers were listed in S4 Table. This plasmid contained a kanamycin cassette, an I-SceI restriction enzyme site, and the duplicated flanking sequences were derived from the region near the KSHV vCyclin arginine 128 coding sequence (approximately 40-bp copy). After PCR amplification, the product was treated with Dpn I to remove the plasmid template. The purified PCR fragments were then introduced into the BAC16-containing GS1783 strain via electroporation, following induction at 42°C for 15 minutes. Recombinant clones were selected on LB plates supplemented with chloramphenicol (12.5 μg/ml) and kanamycin (50 μg/ml) at 32°C. Positive clones were subjected to induction with 1% l-arabinose at 42°C, followed by plating on LB plates containing 1% l-arabinose for secondary recombination. The resulting BAC was subsequently confirmed through Sanger sequencing. The primers used for Sanger sequencing were listed in S4 Table. The BAC16-WT and BAC16-R128K plasmids were isolated using the NucleoBond Xtra Midi Kit. Then, 4 μg plasmids was transfected into iSLK cells using 10 μl of FuGENE HD Transfection Reagent.

### KSHV virion preparation, infection

BAC16-WT and BAC16-R128K cells were seeded in a 12-well plate at a density of 2×10^5^ cells per well. Cells were treated with Dox (4 μg/ml) and NaB (1 mM) for 96 hours. Following treatment, the supernatant from each group was collected and used to infect HEK293T cells. After centrifugal infection for 2 hours at 37°C and 2500 rpm, the medium was removed and replaced with fresh DMEM.

### In vitro methylation analysis

In vitro methylation analysis was carried out as previously described [85, 86]. Briefly, purified GST-vCyclin and PRMT5 proteins were incubated in reaction buffer (250μM Tris-HCl, pH8.0, 25mM MgCl_2_, 100mM KCl) containing methyl group donor SAM (5mM) (Sigma) at 37°C for 2h. The reaction stopped with the SDS sample buffer subjected to SDS-PAGE and immunoblot with the indicated antibody. For the In vitro radioactivity assay, purified GST-vCyclin, GST-PRMT5, and GST-MEP50 were incubated in reaction buffer (250μM Tris-HCl, pH8.0, 25mM MgCl_2_, 100mM KCl) in the presence of 1 μCi of H^3^-labeled SAM at 37°C for 2h. Reactions were spotted onto filters (Whatman) and washed five times with 50 mM NaHCO3 (pH 9.0) before scintillation counting.

### In vitro kinase assays

In vitro kinase assay was carried out as previously described [56, 85]. In brief, the purified GST tagged PRMT5, MEP50, vCyclin, CDK6 and His tagged RB (640-928aa) were incubated in 20μl reaction buffer (50mM Tris-HCl, pH7.5, 20mM MgCl_2_, 1Mm EGTA, 5mM DTT, 100mM Na_3_VO_4_, 0.02%) with 200μM cold ATP at 30°C for 2h. The reaction stopped with the SDS sample buffer was subjected to SDS-PAGE and immunoblot with the indicated antibody.

### Statistical analysis

The statistical analysis was performed through GraphPad Prism software. Data were determined by the unpaired, two-tailed Student’s t-tests. Error bars represent the standard deviation from triplicate samples. ns, P value >0.05; *, P value <0.05; **, P value <0.01; ***, P value < 0.001; ****, P value <0.0001.

## Supporting information

S1 Data Excel spreadsheet containing, in separate sheets, the underlying numerical data and statistical analysis for Figs 2B, 2D, 2F, 2H, 2K, 2M, 3A and 3B, 3D and 3E, 3G, 3H, 3J, 3L, 3M, 4A, 4B, 4E, 4J, 5F, 6D, 6F, 6H, 6I, 7F and S2A, S2B, S2D and S2E, S2G, S2H, S2J, S2L, S2M, S3B, S3D, S4C, S5B, S6E Figs. (XLSX)

S1 Fig PRMT5 expression was declined when LANA was eliminated. (A) SLK cells were infected with or without the wild-type KSHV (KSHV-WT) or the LANA-depleted KSHV (KSHV-LANAstop). The expression of LANA and PRMT5 were measured by western blots.
(TIF)

S2 Fig Knockdown of endogenous PRMT5 inhibits the cell cycle progression and cell proliferation in iSLK.RGB cells. (A) iSLK.RGB cells and SLK (H) cells were transfected with siRNA as indicated. 72 h post-transfection, the knockdown efficiency of PRMT5 was determined by immunoblotting and qPCR analysis. (B) The transcription level of several KSHV genes from iSLK.RGB with knockdown of endogenous PRMT5 was determined by qPCR analysis. (C-D) Flow cytometry was performed to analyze the cell cycle distribution in iSLK.219 cells. Since iSLK.RGB cells contain red fluorescence, which affects the detection of the propidium iodide (PI) dye, iSLK.219 cells were used here instead of iSLK.RGB cells for flow cytometry experiments. Endogenous PRMT5 in iSLK.219 cells were knocked down with two specific siRNAs (siPRMT5#1 and siPRMT5#2) or transfected cells with a negative control siRNA (siNC). 72 hours post-transfection, cells were fixed and stained for flow cytometry analysis. (E-G) The cell proliferation of iSLK.RGB was measured by CCK8 assays (E) and EdU assays (F-G) as previously described. Representative immunofluorescence images show the expression of EdU. Scale bars represent 100μm. (I-J) Flow cytometry was performed to analyze the cell cycle distribution in SLK cells with transfected siNC or siPRMT5#1 or siPRMT5#2 for 72 hours. (K-M) The cell proliferation of SLK cells was measured by CCK8 assays (M) and EdU assays (K-L). Representative immunofluorescence images show the expression of EdU. Scale bars represent 100μm. Representative results from three biological replicates are presented. Error bars indicate SD. Data were analyzed with Student’s multiple t-tests (*p < 0.05, ***p< 0.001, ****p< 0.0001).
(TIF)

S3 Fig Pharmaceutical inhibition (using EPZ015666) of PRMT5 inhibits cell proliferation. (A) iSLK.RGB cells were treated with EPZ015666 (50μM) for 48 hours, followed by immunoblot analysis of the PRMT5 protein. (B) The cell proliferation of iSLK.RGB under treatment with EPZ015666 was assessed using CCK-8 assays. (C) The EDU assay was used to determine the cell proliferation of iSLK.RGB treated with EPZ015666 at the indicated time points. (D) The EDU data from (C) was analyzed by imageJ. Representative results from three biological replicates are presented. Error bars indicate SD. Data were analyzed with Student’s multiple t-tests (*p < 0.05, ***p< 0.001, ****p< 0.0001).
(TIF)

S4 Fig PRMT5 fails to stabilize CyclinD2 or mediate its di-methylation. (A) HEK293T cells were transfected with HA-cyclinD2 alone or together with HA-PRMT5 or together with HA-PRMT5-E444Q. After 36h transfection, the cells were lysed and analyzed by western blotting with indicated antibodies. (B) HEK293T cells were transfected with or without HA-PRMT5. 24h post-transfection, the cells were incubated with 100μg/ml CHX for different time points, then lysed and analyzed by immunoblotting with indicated antibodies. (C) The relative protein abundances of cyclinD2 from immunoblots (B) were quantified by band intensities. Representative results from three biological replicates were presented. (D) iSLK.RGB cells were transfected with two PRMT5 siRNAs. At 72 h posttransfection, lysates of cells were subjected to western blot analysis with human anti-cyclinD2 and KSHV anti-vCyclin antibodies. (E) Purified PRMT5/ MEP50, and vCyclin or cyclinD2 proteins were incubated with SAM for in vitro methylation reaction. Then the reaction buffer was subjected to SDS-PAGE and immunoblotted with anti-SYM10 antibodies.
(TIF)

S5 Fig The Arg 128 residue, rather than other Arg residues of vCyclin, is critical for the stabilization of vCyclin. (A) vCyclin-WT and vCyclin-R51K plasmids were transfected into HEK293T cells with PRMT5 for 24 h, and then the cells were treated with CHX at different time points as indicated. The expression of vCyclin was detected by immunoblotting. (B) The relative protein abundances from immunoblots (A) were quantified by band intensities.
(TIF)

S6 Fig Construction and identification of KSHV BAC16-vCyclin-R128K. (A) BACmids construction. Scheme of two-step red recombination for the construction of the KSHV BAC16-vCyclin-R128K. (B) Sanger sequences for the verification of recombinant BACmids. (C) After transfecting BAC16-WT and BAC16-R128K into iSLK. puro cells and the positive cells (green fluorescence) were sorted by flow cytometry. (D) Positive KSHV BAC16 cells was detected by fluorescence microscopy according to green fluorescence. Scale bars represent 100μm. (E) Equal numbers of BAC16-WT and BAC16-R128K live cells were plated and inducted with Dox and sodium butyrate NaB for 96 hours. Then the viral supernatant (2ml) was used to infected HEK293T cells. At 30 h post-infection, HEK293T cells were collected for extracting viral genomic DNA. Then the relative DNA levels of KSHV was detected by qPCR.
(TIF)

S7 Fig Comparison of the impact of PRMT5 and PEST motif on vCyclin Stability. Schematic diagram of the vCyclin/cyclinD2 chimeric protein. The carboxyl-terminal nucleotide sequence of cyclin D2, which includes the PEST motif, was joined to the 3′-terminal of the nucleotide sequence of vCyclin. (B) HEK293T cells were co-transfected with HA-PRMT5 and Flag-vCyclin or Flag-vCyclin-PEST for 24 h and then cells were treated with CHX at different time points as indicated. The expression of vCyclin and vCyclin-PEST were analyzed by immunoblotting with indicated antibodies.
(TIF)

S1 Table Proteins interacted with vCyclin in HEK293T.219 cells. (XLSX)

S2 Table Proteins interacted with vCyclin in HEK293T cells. (XLSX)

S3 Table Twelve candidate binding factors of vCyclin identified through Venn diagram intersections. (XLSX)

S4 Table Primers for PCR amplification. (DOCX)

S5 Table Primers for qPCR. (DOCX)
