# Supplementary figures and images for: Methylation of KSHV vCyclin by PRMT5 contributes to cell cycle progression and cell proliferation
Source: PLoS Pathog. 2024 Sep 10;20(9):e1012535. doi: 10.1371/journal.ppat.1012535 (PMC11421797; doi:10.1371/journal.ppat.1012535)

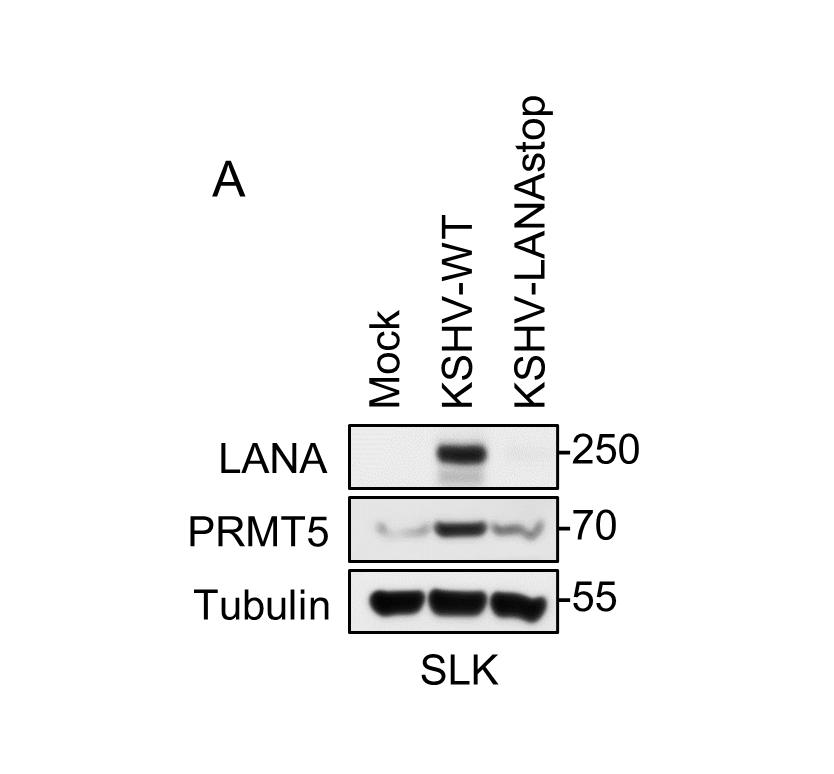

Supplement: S1 Fig — (A) SLK cells were infected with or without the wild-type KSHV (KSHV-WT) or the LANA-depleted KSHV (KSHV-LANAstop). The expression of LANA and PRMT5 were measured by western blots. (TIF) [file ppat.1012535.s002.tif]

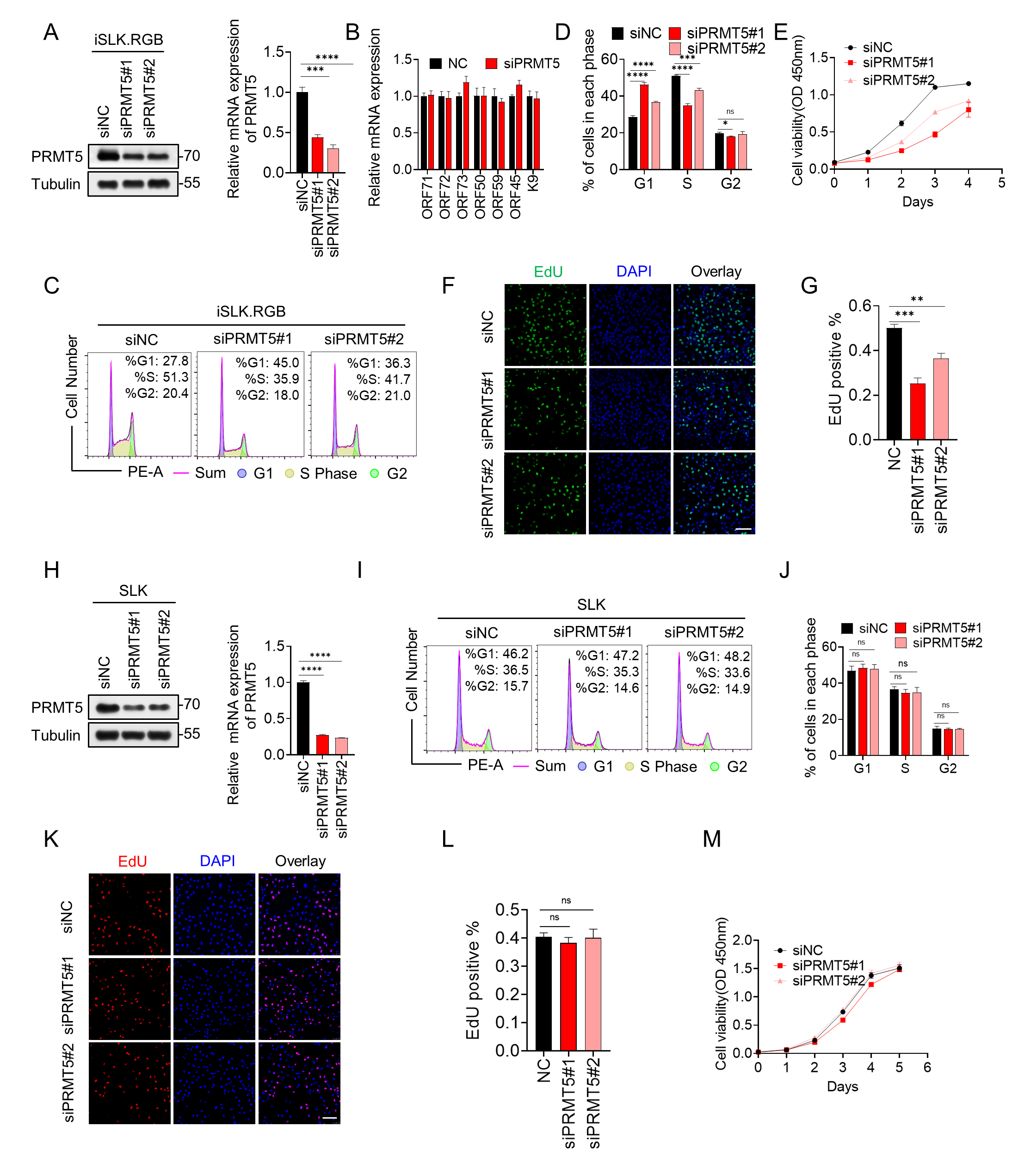

Supplement: S2 Fig — (A) iSLK.RGB cells and SLK (H) cells were transfected with siRNA as indicated. 72 h post-transfection, the knockdown efficiency of PRMT5 was determined by immunoblotting and qPCR analysis. (B) The transcription level of several KSHV genes from iSLK.RGB with knockdown of endogenous PRMT5 was determined by qPCR analysis. (C-D) Flow cytometry was performed to analyze the cell cycle distribution in iSLK.219 cells. Since iSLK.RGB cells contain red fluorescence, which affects the detection of the propidium iodide (PI) dye, iSLK.219 cells were used here instead of iSLK.RGB cells for flow cytometry experiments. Endogenous PRMT5 in iSLK.219 cells were knocked down with two specific siRNAs (siPRMT5#1 and siPRMT5#2) or transfected cells with a negative control siRNA (siNC). 72 hours post-transfection, cells were fixed and stained for flow cytometry analysis. (E-G) The cell proliferation of iSLK.RGB was measured by CCK8 assays (E) and EdU assays (F-G) as previously described. Representative immunofluorescence images show the expression of EdU. Scale bars represent 100μm. (I-J) Flow cytometry was performed to analyze the cell cycle distribution in SLK cells with transfected siNC or siPRMT5#1 or siPRMT5#2 for 72 hours. (K-M) The cell proliferation of SLK cells was measured by CCK8 assays (M) and EdU assays (K-L). Representative immunofluorescence images show the expression of EdU. Scale bars represent 100μm. Representative results from three biological replicates are presented. Error bars indicate SD. Data were analyzed with Student’s multiple t-tests (*p < 0.05, ***p< 0.001, ****p< 0.0001). (TIF) [file ppat.1012535.s003.tif]

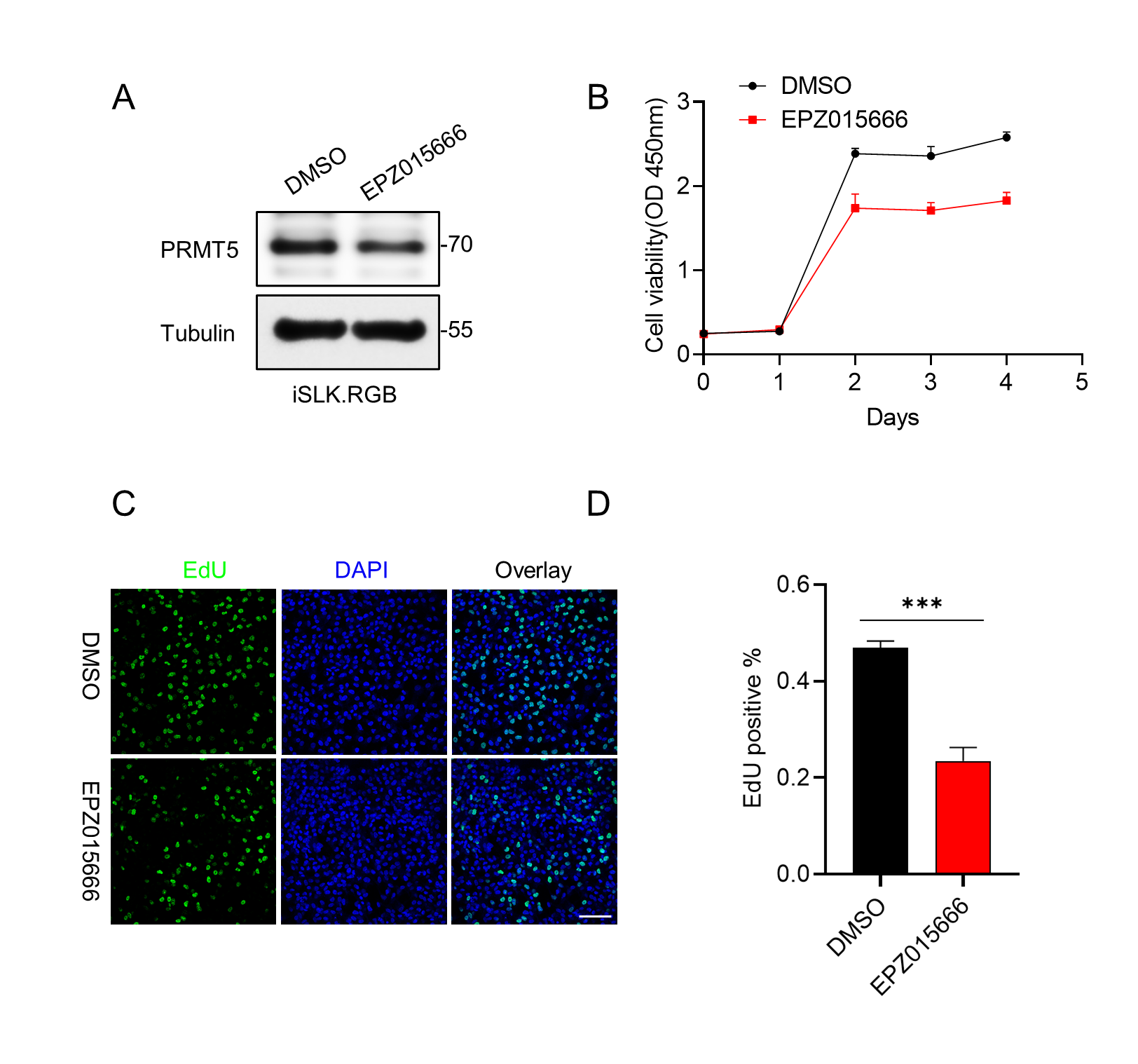

Supplement: S3 Fig — (A) iSLK.RGB cells were treated with EPZ015666 (50μM) for 48 hours, followed by immunoblot analysis of the PRMT5 protein. (B) The cell proliferation of iSLK.RGB under treatment with EPZ015666 was assessed using CCK-8 assays. (C) The EDU assay was used to determine the cell proliferation of iSLK.RGB treated with EPZ015666 at the indicated time points. (D) The EDU data from (C) was analyzed by imageJ. Representative results from three biological replicates are presented. Error bars indicate SD. Data were analyzed with Student’s multiple t-tests (*p < 0.05, ***p< 0.001, ****p< 0.0001). (TIF) [file ppat.1012535.s004.tif]

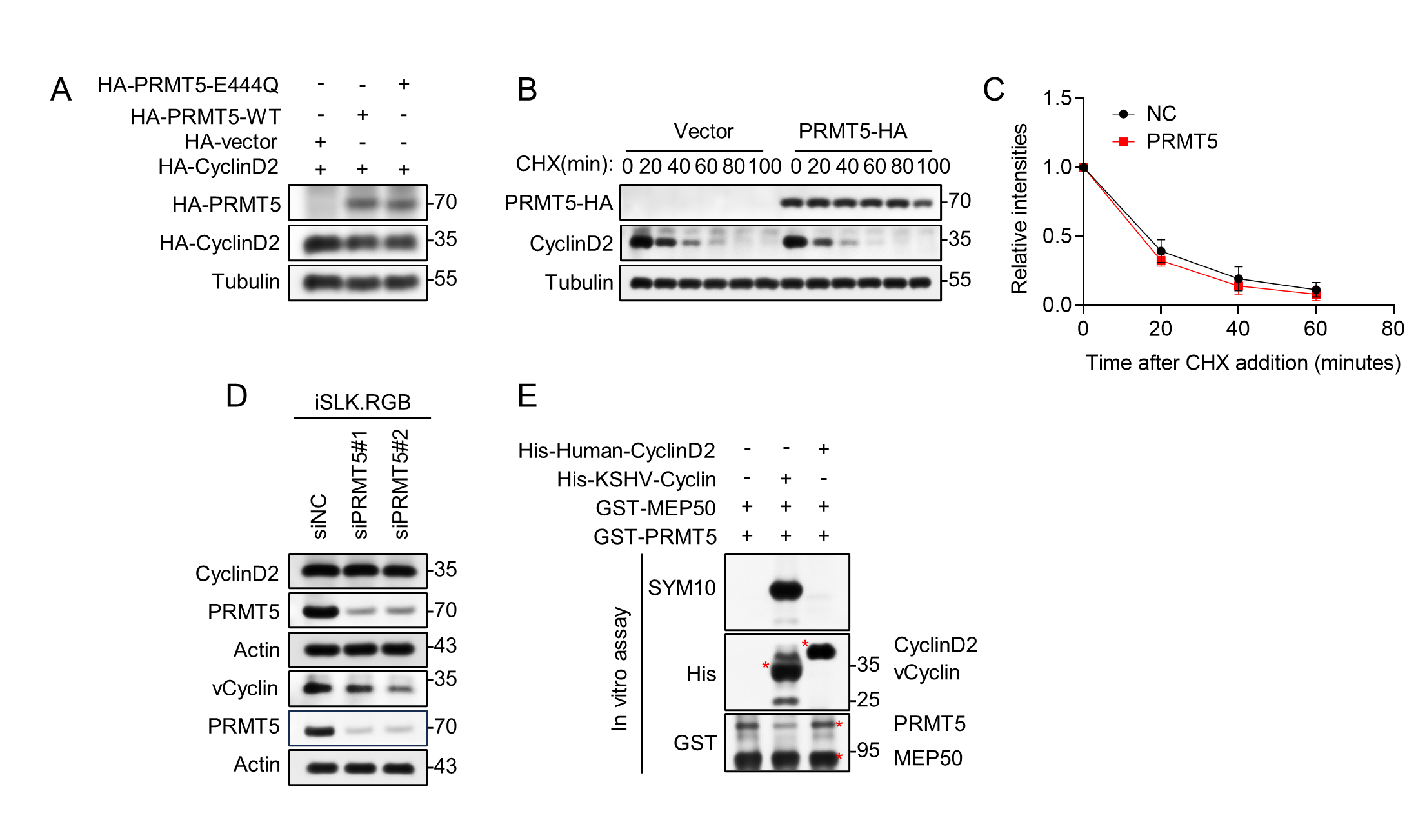

Supplement: S4 Fig — (A) HEK293T cells were transfected with HA-cyclinD2 alone or together with HA-PRMT5 or together with HA-PRMT5-E444Q. After 36h transfection, the cells were lysed and analyzed by western blotting with indicated antibodies. (B) HEK293T cells were transfected with or without HA-PRMT5. 24h post-transfection, the cells were incubated with 100μg/ml CHX for different time points, then lysed and analyzed by immunoblotting with indicated antibodies. (C) The relative protein abundances of cyclinD2 from immunoblots (B) were quantified by band intensities. Representative results from three biological replicates were presented. (D) iSLK.RGB cells were transfected with two PRMT5 siRNAs. At 72 h posttransfection, lysates of cells were subjected to western blot analysis with human anti-cyclinD2 and KSHV anti-vCyclin antibodies. (E) Purified PRMT5/ MEP50, and vCyclin or cyclinD2 proteins were incubated with SAM for in vitro methylation reaction. Then the reaction buffer was subjected to SDS-PAGE and immunoblotted with anti-SYM10 antibodies. (TIF) [file ppat.1012535.s005.tif]

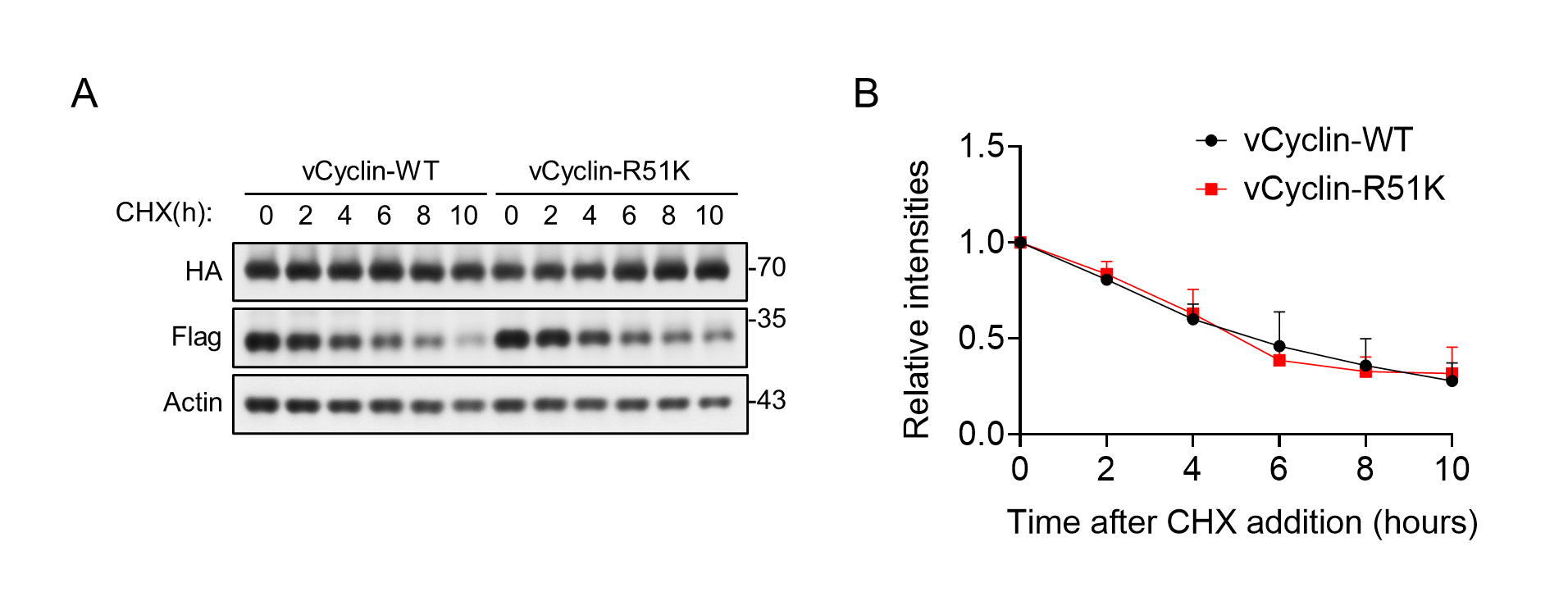

Supplement: S5 Fig — (A) vCyclin-WT and vCyclin-R51K plasmids were transfected into HEK293T cells with PRMT5 for 24 h, and then the cells were treated with CHX at different time points as indicated. The expression of vCyclin was detected by immunoblotting. (B) The relative protein abundances from immunoblots (A) were quantified by band intensities. (TIF) [file ppat.1012535.s006.tif]

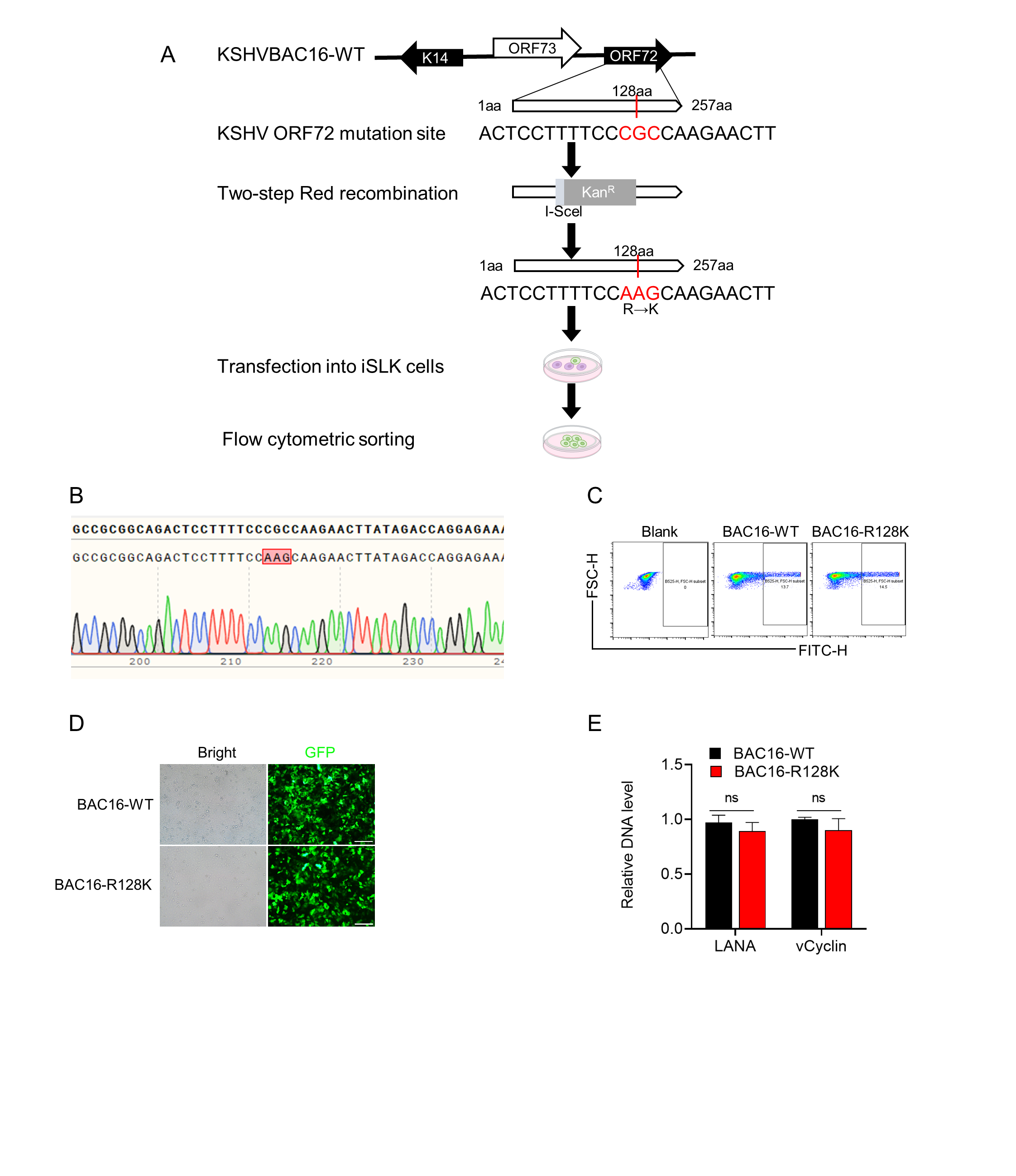

Supplement: S6 Fig — (A) BACmids construction. Scheme of two-step red recombination for the construction of the KSHV BAC16-vCyclin-R128K. (B) Sanger sequences for the verification of recombinant BACmids. (C) After transfecting BAC16-WT and BAC16-R128K into iSLK. puro cells and the positive cells (green fluorescence) were sorted by flow cytometry. (D) Positive KSHV BAC16 cells was detected by fluorescence microscopy according to green fluorescence. Scale bars represent 100μm. (E) Equal numbers of BAC16-WT and BAC16-R128K live cells were plated and inducted with Dox and sodium butyrate NaB for 96 hours. Then the viral supernatant (2ml) was used to infected HEK293T cells. At 30 h post-infection, HEK293T cells were collected for extracting viral genomic DNA. Then the relative DNA levels of KSHV was detected by qPCR. (TIF) [file ppat.1012535.s007.tif]

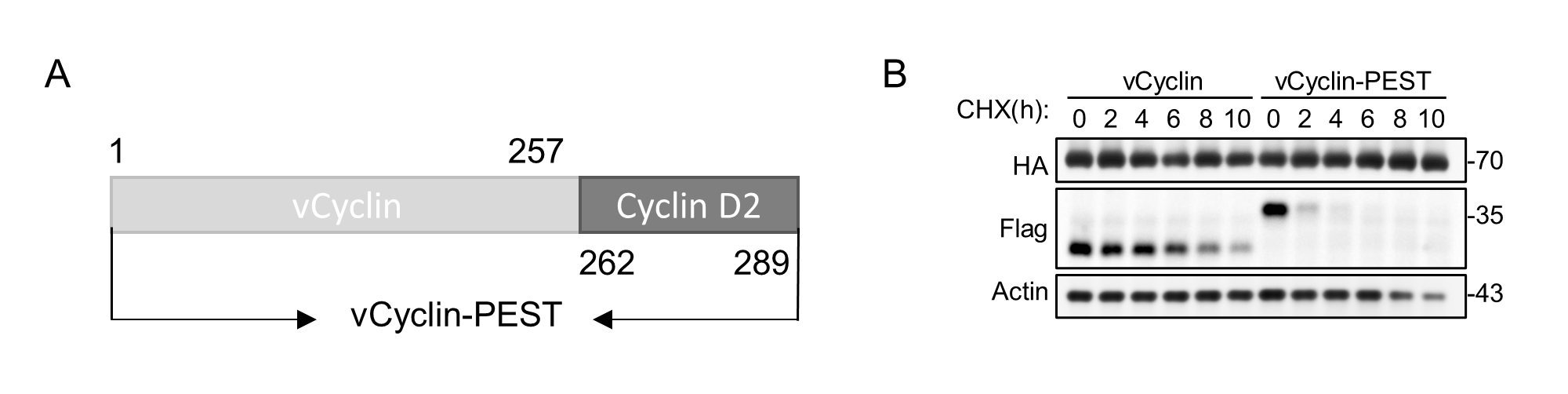

Supplement: S7 Fig — Schematic diagram of the vCyclin/cyclinD2 chimeric protein. The carboxyl-terminal nucleotide sequence of cyclin D2, which includes the PEST motif, was joined to the 3′-terminal of the nucleotide sequence of vCyclin. (B) HEK293T cells were co-transfected with HA-PRMT5 and Flag-vCyclin or Flag-vCyclin-PEST for 24 h and then cells were treated with CHX at different time points as indicated. The expression of vCyclin and vCyclin-PEST were analyzed by immunoblotting with indicated antibodies. (TIF) [file ppat.1012535.s008.tif]
