# Supplementary material for: Methylation of KSHV vCyclin by PRMT5 contributes to cell cycle progression and cell proliferation
Source: PLoS Pathog. 2024 Sep 10;20(9):e1012535. doi: 10.1371/journal.ppat.1012535 (PMC11421797; doi:10.1371/journal.ppat.1012535)
Supplement: S4 Table — (DOCX) [file ppat.1012535.s012.docx]

**S4 Table. Primers for PCR amplification**

| Primer name | Sequence of oligonucleotide (5’-3’) |
| --- | --- |
| pCDH-FLAG-vCyclin-F | cgagctcaagcttcgaattcGCCACCATGGCAACTGCCAATA |
| pCDH-FLAG-vCyclin-R | tgagggtggctccaggatccATAGCTGTCCAGAATGCGCAGA |
| pCDH-FLAG-PRMT5-F | cgagctcaagcttcgaattcGCCACCATGGCGGCGATGGCGG |
| pCDH-FLAG-PRMT5-R | tgagggtggctccaggatccGAGGCCAATGGTATATGAGCGG |
| pCDH-FLAG-vCyclin-R20K-F | ACGCTATGTGAGGATAAGATCTTTTACA |
| pCDH-FLAG-vCyclin-R20K-R | CTTATCCTCACATAGCGTGGGATCCAGA |
| pCDH-FLAG-vCyclin-R31K-F | CTTGAAATTGAGCCGAAGTTTTTAACTT |
| pCDH-FLAG-vCyclin-R31K-R | CTTCGGCTCAATTTCAAGAATATTGTAA |
| pCDH-FLAG-vCyclin-R51K-F | CTTACTTCGCATATGAAGAAGTTACTGG |
| pCDH-FLAG-vCyclin-R51K-R | CTTCATATGCGAAGTAAGAGATTGTTGA |
| pCDH-FLAG-vCyclin-R81K-F | TTAATCTTTTGGACAAACTCCTACTT |
| pCDH-FLAG-vCyclin-R81K-R | TTGTCCAAAAGATTAAGGGCCAACGC |
| pCDH-FLAG-vCyclin-R108K-F | TGGCCAGTAAGCTCAAAAGCCTCACG |
| pCDH-FLAG-vCyclin-R108K-R | TTGAGCTTACTGGCCACTAACAGGCA |
| pCDH-FLAG-vCyclin-R128K-F | GCAGACTCCTTTTCCAAGCAAGAACTTA |
| pCDH-FLAG-vCyclin-R128K-R | CTTGGAAAAGGAGTCTGCCGCGGCATAG |
| pCDH-FLAG-vCyclin-R145K-F | GAGAAGTTGGCGTGGAAGACAGAGGCAG |
| pCDH-FLAG-vCyclin-R145K-R | CTTCCACGCCAACTTCTCAAGGAGTTCT |
| pCDH-FLAG-vCyclin-R252K-F | TCGGACTTTGATCTGAAGATTCTGGACA |
| pCDH-FLAG-vCyclin-R252K-R | CTTCAGATCAAAGTCCGAAACAGATGTT |
| pCDNA3.1-HA-PRMT5-F | agtccagtgtggtggaattcGCCACCATGGCGGCGATGGCGG |
| pCDNA3.1-HA-PRMT5-R | acgtcgtatgggtatctagaGAGGCCAATGGTATATGAGCGG |
| pCDNA3.1-HA-PRMT5-E444Q-F | TCATTTGCTGACAATCAATTGTCGCCTGAGT |
| pCDNA3.1-HA-PRMT5-E444Q-R | GATTGTCAGCAAATGAGCCCAGAAGC |
| pCDNA3.1-HA-vCyclin-F | agtccagtgtggtggaattcGCCACCATGGCAACTGCCAATA |
| pCDNA3.1-HA-vCyclin-R | acgtcgtatgggtatctagaATAGCTGTCCAGAATGCGCAGA |
| pCDNA3.1-HA-MEP50-F | agtccagtgtggtggaattcGCCACCATGCGGAAGGAAACCC |
| pCDNA3.1-HA-MEP50-R | acgtcgtatgggtatctagaCTCAGTAACACTTGCAGGTCCA |
| pCDNA3.1-HA-CDK6-F | agtccagtgtggtggaattcGCCACCATGGAGAAGGACGGCC |
| pCDNA3.1-HA-CDK6-R | acgtcgtatgggtatctagaGGCTGTATTCAGCTCCGAGGTG |
| pCDNA3.1-HA-cyclinD2-F | agtccagtgtggtggaattcGCCACCATGGAGCTGCTGTGCC |
| pCDNA3.1-HA-cyclinD2-R | acgtcgtatgggtatctagaCAGGTCGATATCCCGCACGTCT |
| pGEX-4T-1-vCyclin-F | cgcgtggatccccggaattcATGGCAACTGCCAATAACCCGC |
| pGEX-4T-1-vCyclin-R | tcacgatgcggccgctcgagTTAATAGCTGTCCAGAATGCGC |
| pGEX-4T-1-PRMT-F | cgcgtggatccccggaattcATGGCGGCGATGGCGGTCGGGG |
| pGEX-4T-1-PRMT-R | tcacgatgcggccgctcgagCTAGAGGCCAATGGTATATGAG |
| pGEX-4T-1-MEP50-F | cgcgtggatccccggaattcATGCGGAAGGAAACCCCACCCC |
| pGEX-4T-1-MEP50-R | tcacgatgcggccgctcgagCTACTCAGTAACACTTGCAGGT |
| pGEX-4T-1-CDK6-F | cgcgtggatccccggaattcATGGAGAAGGACGGCCTGTGCC |
| pGEX-4T-1-CDK6-R | tcacgatgcggccgctcgagTCAGGCTGTATTCAGCTCCGAG |
| pET-30a-His-RB (640-928aa)-F | ctgatatcggatccgaattcAAGCCATTGAAATCTACCTCTC |
| pET-30a-His-RB (640-928aa)-R | tggtggtggtggtgctcgagTTTCTCTTCCTTGTTTGAGGTA |
| pET-30a-His-cyclinD2-F | ctgatatcggatccgaattcATGGAGCTGCTGTGCCACGAGG |
| pET-30a-His-cyclinD2-R | tggtggtggtggtgctcgagCAGGTCGATATCCCGCACGTCT |
| pGL3-Enhancer-PRMT5-F | tttctctatcgataggtaccCGCCACCACGTCTGGCTAAATT |
| pGL3-Enhancer-PRMT5-R | tcgcagatctcgagcccgggTCCGCTCGTGGAGGTCCGGCCC |
| BAC16-R128K-F | TACCAGTTCACTTTGCTATGCCGCGGCAGACTCCTTTTCCAAGCAAGAACTTATAGACCAG AGGATGACGACGATAAGTAGGG |
| BAC16-R128K-R | TTCTCAAGGAGTTCTTTCTCCTGGTCTATAAGTTCTTGCTTGGAAAAGGAGTCTGCCGCGGCAACCAATTAACCAATTCTGATTAG |
| BAC16-Sangerseq-F | CCTAGAACCTAACGTGGTCGC |
| BAC16-Sangerseq-R | AGCTCCAATTAATTGGGCCA |
| vCyclin-PEST-F | GCGCATTCTGGACAGCTATCGTCAGGACCAACGTGACGG |
| vCyclin-PEST-R | ctcgaactgagggtggctccaCAGGTCGATATCCCGCACGT |
