# Supplementary material for: Methylation of KSHV vCyclin by PRMT5 contributes to cell cycle progression and cell proliferation
Source: PLoS Pathog. 2024 Sep 10;20(9):e1012535. doi: 10.1371/journal.ppat.1012535 (PMC11421797; doi:10.1371/journal.ppat.1012535)
Supplement: S5 Table — (DOCX) [file ppat.1012535.s013.docx]

**S5 Table. Primers for qPCR analysis**

| Primer name | Sequence of oligonucleotide (5’-3’) |
| --- | --- |
| qPCR-PRMT5 -F | CTGTCTTCCATCCGCGTTTCA |
| qPCR-PRMT5 -R | GCAGTAGGTCTGATCGTGTCTG |
| qPCR-Actin -F | CATGTACGTTGCTATCCAGGC |
| qPCR-Actin -R | CTCCTTAATGTCACGCACGAT |
| qPCR-RTA -F | CGTGTAGAGATTCAACGGCG |
| qPCR-RTA -R | AAGAGGTACCAGGTGTCGTG |
| qPCR-vCyclin -F | GCTGATAATAGAGGCGGGCAATGAG |
| qPCR-vCyclin -R | GTTGGCGTGGCGAACAGAGGCAGTC |
| qPCR-LANA -F | GCTTGGTCCGGCTGACTTAT |
| qPCR-LANA -R | TGCAGTACCGCCCATGG |
| qPCR-K9-F | GTCTCTGCGCCATTCAAAAC |
| qPCR-K9-R | CCGGACACGACAACTAAGAA |
| qPCR-vFlip -F | GGATGCCCTAATGTCAATGC |
| qPCR-vFlip-R | GGCGATAGTGTTGGGAGTGT |
| qPCR-ORF59 -F | CGAGTCTTCGCAAAAGGTTC |
| qPCR-ORF59 -R | CGAGTCTTCGCAAAAGGTTC |
| qPCR-ORF45 -F | CCTTTATCTCACTTGCGCCC |
| qPCR-ORF45 -R | TCGTCGTCTGAAGGTGAGAG |
| qPCR-Tubulin-F | TCCATGAAGGAGGTCGATGA |
| qPCR-Tubulin-R | CAGACGGCTGTCTTGACATT |
